# Interleukin (IL)-17/IL-36 axis participates to the crosstalk between endothelial cells and keratinocytes during inflammatory skin responses

**DOI:** 10.1371/journal.pone.0222969

**Published:** 2020-04-30

**Authors:** Laura Mercurio, Cristina M. Failla, Lorena Capriotti, Claudia Scarponi, Francesco Facchiano, Martina Morelli, Stefania Rossi, Gianluca Pagnanelli, Cristina Albanesi, Andrea Cavani, Stefania Madonna

**Affiliations:** 1 Laboratory of Experimental Immunology, IDI-IRCCS, Rome, Italy; 2 Department of Oncology and Molecular Medicine, Istituto Superiore di Sanità (ISS), Rome, Italy; 3 1st Dermatology Division, IDI-IRCCS, Rome, Italy; 4 National Institute for Health, Migration and Poverty (NIHMP), Rome, Italy; INSERM, FRANCE

## Abstract

In inflammatory skin conditions, such as psoriasis, vascular enlargement is associated with endothelial cell proliferation, release of cytokines and adhesion molecule expression. Interleukin (IL)-17A is a pro-inflammatory cytokine mainly secreted by T helper-17 cells that is critically involved in psoriasis pathogenesis. IL-36α, IL-36β and IL-36γ are also inflammatory cytokines up-regulated in psoriasis and induced by various stimuli, including IL-17A. In this study, we found that human keratinocytes are the main source of IL-36, in particular of IL-36γ. This cytokine was strongly induced by IL-17A and, together with IL-17A, efficiently activated human dermal microvascular endothelial cells (HDMECs), which expressed both IL-17 and IL-36 receptors. Both IL-36γ and IL-17A induced cell proliferation through specific molecular cascades involving ERK1/2 only or ERK1/2, STAT3 and NF-κB, respectively. We highlighted the intense IL-17A- and IL-36γ -dependent interplay between keratinocytes and HDMECs, likely active in the psoriatic lesions and leading to the establishment of a cytokine network responsible for the development and maintenance of the inflamed state. IL-17A or IL-36γ showed in HDMECs a synergic activity with TNF-α by potently inducing inflammatory cytokine/chemokine release and ICAM-1 expression. We also investigated the involvement of IL-36γ and VEGF-A, substantially reduced in lesional skin of psoriatic patients pharmacologically treated with the anti-IL-17A antibody Secukinumab. Importantly, keratinocyte-derived IL-36γ represented an additional pro-angiogenic mediator of IL-17A. We observed that keratinocyte-derived VEGF-A influenced proliferation but did not act on expression of adhesion molecules in HDMECs. On the other hand, inhibition of IL-36γ released by IL-17A-treated keratinocytes impaired either proliferation or ICAM-1 expression both in HDMECs and in an *in vivo* murine model of psoriasis. Taken together, our data demonstrated that IL-17A and IL-36γ are highly involved in endothelial cells/keratinocytes crosstalk in inflammatory skin conditions.

## Introduction

Blood and lymphatic vessels have a major role in skin inflammation [1]. In chronic inflammatory disorders, such as psoriasis, vascular enlargement is associated to vessel hyper-permeability and endothelial cell (EC) proliferation. Vessel morphological changes are evident well before the development of epidermal hyperplasia, even if most pro-angiogenic factors are produced by epidermal keratinocytes themselves [2]. Besides, activated endothelium expresses adhesion molecules and secretes cytokines and chemokines that support leukocyte extravasation and migration into the skin, thus contributing to disease pathogenesis [3]. Under inflammatory conditions, MHC class II^+^ ECs have been also involved in the selective amplification of interleukin (IL)-17-producing CD4^+^ T helper (Th) lymphocytes [4,5]. IL-17 cytokines, in particular IL-17A, are potent proinflammatory cytokines secreted by Th-17 cells and by additional adaptive and innate lymphocytes as well as neutrophils and mast cells [6]. The IL-17 family comprises six members that exert their functions as homodimers with the exception of IL-17A and IL-17F that can form heterodimers. In a similar way, IL-17 cytokines signal via heterodimeric receptors (IL-17R) and IL-17A, IL-17F or IL-17A/IL-17F heterodimers bind to the same receptor composed of IL-17RA and IL-17RC subunits. IL-17RA is ubiquitously expressed in epithelial, hematopoietic cells, fibroblasts and osteoblasts, as well as ECs [7]. However, IL-17 family involvement in EC biological responses is still a controversial issue, especially in inflammatory conditions. Tumors expressing IL-17A show a high vascular density, and IL-17A elicits neovascularization in a rat cornea assay [8]. Some authors reported that IL-17A does not directly affect endothelial cell proliferation *in vitro* [8] but significantly enhances proliferation induced by other angiogenic cytokines such as vascular endothelial growth factor (VEGF)-A [9]. Moreover, IL-17A induces EC migration and tubular structure formation *in vitro* [8]. Other studies reported a direct role of IL-17A in vessel growth *in vitro* and *in vivo*, through activation of both IL-17RA and IL-17RC [10]. Furthermore, Liu *et al*. reported that IL-17A effects on vascular inflammation were not mediated by ECs but rather by pericytes [11].

On ECs and other cell types, most of the IL-17A-induced inflammation depends on its capability to act synergistically with other stimuli. IL-17A and IL-6 together induce ICAM-1 up-regulation in ECs, enhancing monocyte adhesion to vessels [12]. In the case of tumor necrosis factor (TNF)-α, IL-17A stabilizes the mRNA of TNF-activated genes leading to a signal amplification [13]. IL-17 and TNF-α synergistically stimulate cytokine expression in human melanocytes and ECs [14,15]. In human dermal microvascular ECs (HDMECs), IL-17A cooperates with TNF-α in the induction of CSF1/G-CSF and CXCL1/GRO-α [14], whereas in brain ECs IL-17A alone stimulates the release of CCL2/MCP-1 and CXCL1/GRO-α [16]. On an immortalized endothelial cell line, IL-17A was able to stimulate the release of CXCL1/GRO-α, CSF2/GM-CSF and CXCL8/IL-8 [17]. Therefore, literature data are highly variable, considering the different origin of the utilized ECs and the diverse culture conditions.

IL-17A is critically involved in the pathogenesis of psoriasis and several drugs targeting the IL-17A pathway have been developed and are currently used in the clinical practice. IL-17A affects, in particular, keratinocyte immune function, by inducing the release of antimicrobial peptides and chemokines, such as CXCL8/IL-8 and CXCL1/GRO-α, responsible for the accumulation of neutrophils in the early phase of psoriasis inflammation [18]. Among the factors induced by IL-17A, together with TNF-α, there are the IL-36 cytokines that, in turn, augment Th-17 functions, revealing the existence of a feedback loop able to amplify the IL-17 inflammatory signals [19]. IL-36 cytokines belong to the IL-1 family and are highly present in psoriasis, being produced by keratinocytes, macrophages and dendritic cells [20,21]. IL-36α, β and γ initiate a signal cascade that starts with binding to their IL-1Rrp2 receptor and leads to up-regulation of proinflammatory cytokines including IL-6 and CXCL-8/IL-8 [22]. In the psoriasis context, IL-36 cytokines, together with IL-17A, impair keratinocyte differentiation by inducing a proinflammatory skin phenotype [23]. Importantly, IL-36 family impacts on immune response initiation by acting on dendritic and Langerhans cells, recruiting neutrophils, and promoting CD4+ T cell proliferation [24,25]. HDMECs also express IL-36 receptor and respond to IL-36γ stimulation by up-regulating adhesion molecule expression and augmenting chemokine secretion [26]. Less is known about a possible role of IL-36 in the crosstalk between ECs and epidermal keratinocytes.

In this paper, we investigated direct and indirect effects of both IL-17A and IL-36γ on HDMECs, underlining the importance of these cytokines in the crosstalk between keratinocytes and ECs during skin inflammatory processes.

## Materials and methods

### Cell culture

Human keratinocytes were obtained from skin biopsies of healthy donors as previously described [27]. Experiments were carried out on secondary and tertiary cultures and repeated at least three times on different strains. HDMECs were isolated from foreskin of donors as previously described [28] and growth in endothelial cell growth medium (EGM, Lonza, Basel, Switzerland). A pool of HDMECs derived from 4 different healthy donors was used at passages from 2 to 4. In selected experiments, HDMECs were treated with the S3I-201 STAT3 inhibitor (Santa Cruz Biotechnology, Santa Cruz, CA, USA), the SC-514 NF-κB inhibitor (Santa Cruz Biotechnology) or with the PD98059 ERK1/2 inhibitor (Calbiochem, Merck Millipore, Burlington, MA, USA), all at 7.5 μM final concentration in endothelial basal medium (EBM, Lonza) supplemented with 2% fetal bovine serum.

### Western blotting

HDMECs were starved in EBM supplemented with 2% fetal bovine serum for 6 hours and then left untreated or treated with 10 ng/ml IL-17A or 50 ng/ml IL-36γ alone or with the addition of 10 ng/ml TNF-α (R&D Systems, Minneapolis, MN, USA) for 24 hours. Cells were lysed in RIPA buffer [50 mM Tris-HCl (pH 7.4), 150 mM NaCl, 1 mM EGTA, 1% NP-40, 0.25% Na deoxycholate, 0.1% SDS] and 30 μg of the total protein lysate were loaded on a 10% SDS-polyacrylamide gel, transferred to nitrocellulose (Hybond-ECL, GE Bioscience, Chalfont St. Giles, UK) and incubated for 1 hour in Western blocking reagent (Roche Applied Science, Basel, Switzerland). Primary antibodies (anti-human IL-17RA antibody, Cell Signaling Technology, Danvers, MA, USA; anti-human IL-1Rrp2 antibody, Santa Cruz Biotechnology) were used diluted 1:1000 and applied for 18 hours followed by the appropriate horseradish peroxidase-coupled secondary antibody (GE Bioscience). Blots were re-probed with anti-β-actin antibody (diluted 1:4000, Santa Cruz Biotechnology) and stained with Coomassie blue as loading controls as previously described [29]. Detection was performed using the ECL plus detection system (GE Bioscience). The relative intensity of signals was quantified using a GS-710 densitometer (Bio-Rad Laboratories, Hercules, CA, USA). For signaling studies, HDMECs were treated or not with IL-17A (50 ng/ml) or IL-36γ (50 ng/ml) for different times and lysed as aforementioned. Western blotting analyses were performed by using the following primary antibodies: mouse anti-phosphorylated (p)STAT3 (Cell Signaling Technology); mouse anti-pERK1/2 (Santa Cruz Biotechnology); anti-pP65 (Cell Signaling Technology), diluted 1:1000, followed by the appropriate horseradish peroxidase-coupled secondary antibody (GE Bioscience). Blots were re-probed with anti-STAT3, anti-ERK1/2 and anti-P65 antibodies against the not-phosphorylated protein forms (all purchased from Santa Cruz Biotechnologies). The relative intensity of signals was quantified using a GS-710 densitometer (Bio-Rad Laboratories).

### ELISA assay

HDMECs were seeded in 12-well plates, starved in EBM supplemented with 2% fetal bovine serum for 6 hours and then untreated or treated with 10 ng/ml IL-17A, 10 ng/ml TNF-α, 50 ng/ml IL-36γ or a combination of IL-17A or IL-36γ plus TNF-α for 24 hours. Keratinocytes were seeded in 12-well plates and stimulated with 10 ng/ml IL-17 or with a combination of 200 U/ml interferon (IFN)-γ and 50 ng/ml TNF-α or with the three cytokines together for 24 hours in keratinocyte basal medium (KBM, Lonza). Supernatants were collected, cleared by centrifugation, and attached cells were detached and counted by trypan blue colorimetric assay. Duo Set ELISA kits (R&D Systems) were used for IL36α, IL-36β, IL-36γ, VEGF-A, IL-6, CSF3/G-CSF, CXCL10/IP10 and IL-8. For CCL2/MCP1 and CCL5/RANTES detection BD ELISA kits (OptEIA Set, BD Biosciences) were used. Results were normalized to the total number of cells in each sample and were expressed as pg or ng/10^6^ cells. Triplicate wells were used for each condition and experiments were repeated at least three times with comparable results.

### Cell proliferation

Cell proliferation was evaluated by two different methods, namely trypan blue exclusion test and CyQUANT proliferation assay (Invitrogen, Life Technologies, Carlsbad, CA USA). For trypan blue staining, HDMECs were plated in a 6 multi-well at the concentration of 1 x 10^5^ cells/ml. At 40% confluence, cells were starved in EBM supplemented with 2% fetal bovine serum for 4 hours and treated with: i) IL-17A (10 and 50 ng/ml), alone or in combination with 10 ng/ml of TNF-α; ii) IL-36γ (50 ng/ml), alone or in combination with either 10 ng/ml of TNF-α or 10 ng/ml IL-17A, in EBM plus 2% fetal bovine serum; iii) EGM; or iv) untreated. The number of viable cells was determined after 24, 48 and 72 hours at the end of stimulation. To perform the CyQUANT assay, 0.5 × 10^4^ HDMECs were plated in 96-well plates in quadruplicate for each condition. After 1 day, medium was changed with fresh medium in the presence or absence of 7.5 μM of S3I-201, PD98059 or SC-514. After 1-hour pre-incubation, cells were treated with IL-17A (50 ng/ml) or IL-36γ (50 ng/ml) or left untreated. HDMECs were maintained in culture for additional 48 hours and the number of viable cells determined by fluorescence intensity, accordingly to manufacture’s protocol. Fluorescence intensity was detected with Ensight Multilabel Plate Reader (PerkinElmer, Waltham, MA, USA).

In selected experiments, HDMECs were treated with keratinocyte-conditioned medium. Briefly, keratinocytes were seeded at a concentration of 0.4 x 10^5^ cells/ml in 6-well plate and, after reaching about 70% confluence, cells were stimulated for 3 hours with IL-17A (50 ng/ml), alone or in combination with TNF-α (50 ng/ml) in KBM. Medium with stimuli was removed and basal medium was added for 48 hours. Next, HDMECs were seeded in 12-well plates in EGM and 1 day after cells were starved and treated with conditioned medium of keratinocytes, in the presence or not of IL-36RA (R&D Systems, 200 ng/ml), Sunitinib (Pfizer S.r.l., New York, NY, 200 nM) or Bevacizumab (Roche Applied Science, 5 μg/ml). After 48 hours of stimulation, the number of viable cells was determined by CyQUANT Cell Proliferation Assay.

### FACS analysis

Cells were treated as described in the “Cell proliferation" section with: i) IL-17A (10 ng/ml), alone or in combination with 10 ng/ml of TNF-α; ii) IL-36γ (50 ng/ml), alone or in combination with either 10 ng/ml of TNF-α or 10 ng/ml IL-17A, in EBM plus 2% fetal bovine serum; or iii) untreated. HDMEC membrane expression of ICAM-1 was evaluated using allophycocyanin (APC)-conjugated anti-CD54 (ICAM-1) monoclonal Ab (clone 84H10; Immunotech, Marseille, France). VCAM-1 expression and E-selectin expression were detected by using APC-conjugated monoclonal antibodies anti-CD106 (VCAM-1, clone 51-10C9) and anti-CD62E (E-selectin, clone 68-5H11, BD Biosciences) respectively. Cells were analyzed by a FACScan equipped with Cell Quest software (Becton Dickinson, Mountain View, CA, USA). At least three independent experiments were performed.

### Cytokine analysis

HDMECs were starved in EBM supplemented with 2% fetal bovine serum for 6 hours and then untreated or treated with 10 ng/ml IL-17 or TNF-α or both in EBM plus 2% fetal bovine serum for 24 hours. Conditioned medium was collected and analyzed by means of xMAP technology using a X200 Luminex platform (Bio-Plex) equipped with a magnetic workstation. Panel used was the PRO Human Cytokine 27-PLEX for the simultaneous detection of FGF basic, Eotaxin, G-CSF, GM-CSF, IFN-γ, IL-1β, IL-1ra, IL-2, IL-4, IL-5, IL-6, IL-7, IL-8, IL-9, IL-10, IL-12 (p70), IL-13, IL-15, IL-17A, IP-10, MCP-1 (MCAF), MIP-1α, MIP-1β, PDGF-BB, RANTES, TNF-α, and VEGF-A (BioRad) according to the manufacturer's instruction. Data were analyzed using Bio-Plex Software Manager 6.1. Duplicate wells were used for each condition. Coefficient of variation (CV) of measurements of the whole panel was always lower than 10%.

### Imiquimod-induced psoriasiform model

Eight-week-old female BALB/cJ mice (Harlan Laboratories, San Pietro al Natisone, Udine, Italy) were treated for 5 consecutive days with 62.5 mg imiquimod (IMQ) (5% ALDARA cream, Meda AB, Solna, Sweden) [27] and received at the same time daily subcutaneous injections (1 μg/mouse) of human recombinant IL-36RA or control vehicle (1x PBS). On day 5, full thickness skin biopsies of the treated area were collected with an 8-mm biopsy puncher. Skin was fixed in 10% formalin (Sigma-Aldrich, St. Louis, MO, USA) and embedded in paraffin for histopathological analysis. All mouse procedures were carried out in accordance with institutional standard guidelines. The experimental design has been authorized by the Italian Ministry of Health (protocol n. SA-IDI-13-CA-1), five animals were used for each experimental condition.

### Patient samples

Six-mm punch skin biopsies of three patients with mild-to-severe chronic plaque psoriasis undergoing to pharmacological treatment with the anti-IL-17A antibody Secukinumab (Cosentyx, Novartis Farma S.p.A., subcutaneous injection of 300 mg, once a week after an induction phase) were analyzed by immunohistochemistry. For each patient, biopsies were taken before treatment and after an 8-week treatment from lesional skin (LS). Patients received information and gave their consent to participate to the study. The latter was approved by IDI-IRCCS Ethical Committee (IDI-IMM-IL36pso) and performed in accordance with the Helsinki Declaration. Skin samples were fixed in 10% formalin and embedded in paraffin for immunohistochemical analysis.

### Immunohistochemistry

Five-μm sections were dewaxed and rehydrated. After quenching endogenous peroxidase, achieving antigen retrieval and blocking nonspecific binding sites, sections were incubated with the anti-human VEGF-A mouse monoclonal antibody (Beckton Dickinson), at a concentration of 5 μg/ml, anti-mouse PECAM/CD31 rabbit polyclonal antibody (Abcam), at a concentration of 40 μg/ml and anti-mouse CD-54/ICAM-1 hamster monoclonal antibody (BD Pharmingen) at a concentration of 1.25 μg/ml. Secondary biotinylated monoclonal Abs and staining kits were obtained from Vector Laboratories. Immunoreactivity was visualized with peroxidase reaction using 3-amino-9-ethylcarbazole (AEC, Vector Laboratories, Burlingame, CA) in H_2_O_2_ and specimen counterstained with hematoxylin. As a negative control, primary Abs were omitted or replaced with an irrelevant isotype-matched mAb. Stained sections were analyzed with the AxioCam digital camera attached to the Axioplan 2 microscope (Carl Zeiss AG, Oberkochen, Germany). VEGF-A staining intensity was evaluated by a semiquantitative, four-stage scoring system, ranging from negative (0) to strong immunoreactivity (4+). Positive cells for ICAM-1 and CD31 were directly counted. Every quantification was performed on three fields per sample by two independent observers, blinded to the status of the specimens.

### Statistical analysis

Statistical analysis was performed using parametric tests, i.e. Student’s t- and ANOVA one-way tests for the analysis of normally distributed data. For data set that did not follow the parameters of normal distribution curve, non-parametric tests, i.e. Kruskal-Wallis and Mann-Whitney tests were used. Since the different tests gave similar results, only data from one of the two tests used has been reported, as indicated in each figure legend. Statistically significant differences were defined as p<0.05. Post-hoc comparisons were applied to data analyzed with ANOVA one-way test or Kruskal Wallis analysis; Tukey’s test or Dunn test were used, respectively. All analyses were conducted by using GraphPad prism Software (La Jolla, CA, USA).

## Results

In order to investigate the role played by IL-17A and IL-36 cytokines in the activation of skin EC, expression of IL-17 and IL-36 receptors by HDMECs was analyzed, together with possible modulation of this expression by inflammatory cytokines present in the psoriasis microenvironment, such as TNF-α. As shown in S1A Fig and previously reported [11,14], HDMECs expressed both IL-17RA and IL-1Rrp2 and this expression was not significantly influenced by IL-17A and IL-36γ, alone or in combination with TNF-α. Differently from IL-17 isoforms that are mainly produced by leukocytes, IL-36 cytokines are expressed by both HDMECs and keratinocytes [21,29]. HDMECs produced substantial amount of two of the three IL-36 isoforms with their release augmenting upon IL-17A or TNF-α stimulation and even more with their combination (S1B Fig). However, IL-36 levels were lower than those secreted by human keratinocytes (S1C Fig), especially for IL-36γ amounts [29]. Therefore, our experiments were performed with IL-36γ only.

**Fig 1:**
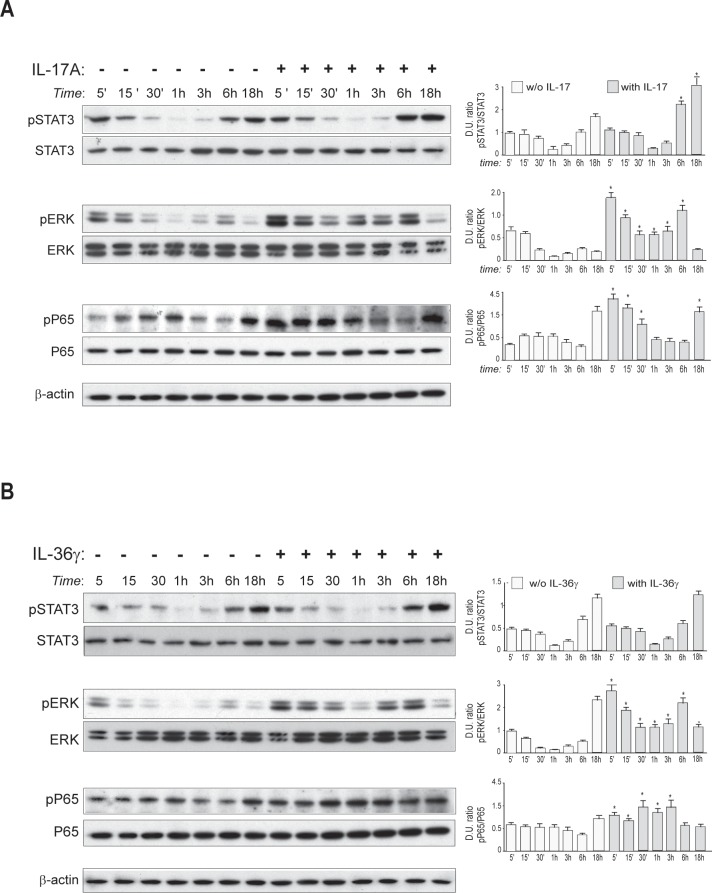
Intracellular signaling induced by IL-17A and IL-36γ in HDMECs. A and B. Protein extracts were obtained from HDMECs treated with IL-17A (A) or IL-36γ (B) for the indicated time points and subjected to Western blotting analysis to detect STAT3, ERK1/2 and P65 phosphorylation. Filters were re-probed with anti-STAT3, -ERK1/2 and -P65 antibodies, whereas β-actin levels were detected as a loading control. One representative experiment out of three performed is shown. Graphs in (A) and (B) show densitometric values of the protein levels obtained in three independent Western blotting analyses. Data are expressed as mean ± SD of the ratio of the Densitometric Units (D.U.) between each value of the indicated phosphorylated versus unphosphorylated proteins; p*≤0.05 using a Student's *t* test between treated and untreated cells.

It is known that binding of IL-17A to its receptor induces the intracellular pathways mediated by NF-κB and p38/MAPK in several cell types [30]. As shown in Fig 1A, we found that in HDMECs IL-17A induced the phosphorylation of the transcription factor STAT3 at late time-points of stimulation (6–18 hours). Additionally, IL-17A had a dual effect on ERK1/2 phosphorylation (Fig 1A) that was up-regulated rapidly after a 5-min treatment (early activation) with IL-17A and gradually declined after 15 min, with levels remaining higher than those observed in untreated cells. After 6 hours (late activation), ERK1/2 phosphorylation returned to be high and decreased thereafter (Fig 1A). Finally, consistently with other reports, IL-17A induced in HDMECs the early phosphorylation of P65, a transcription factor of the NF-κB complex [30]. Differently from IL-17A, IL-36γ did not activate the phosphorylation of STAT3, whereas it strongly induced the phosphorylation of P65, as previously reported [26], and, at a lower extent, that of ERK1/2 (Fig 1B).

We next investigated whether IL-17A or IL36γ could directly influence HDMEC proliferation and if activation of either STAT3, ERK1/2 or P65 was involved in such a process.

We analyzed HDMECs proliferation and found that IL17-A significantly promoted cell proliferation in a dose-response manner at 48 and 72 hours of treatment, as compared to cultures grown in EBM (S2A Fig). Similarly, IL-36γ at the concentration of 50 ng/ml significantly promoted HDMEC proliferation even if less efficiently than IL-17A, and the association of the two cytokines did not further enhance cell proliferation (S2B Fig).

**Fig 2:**
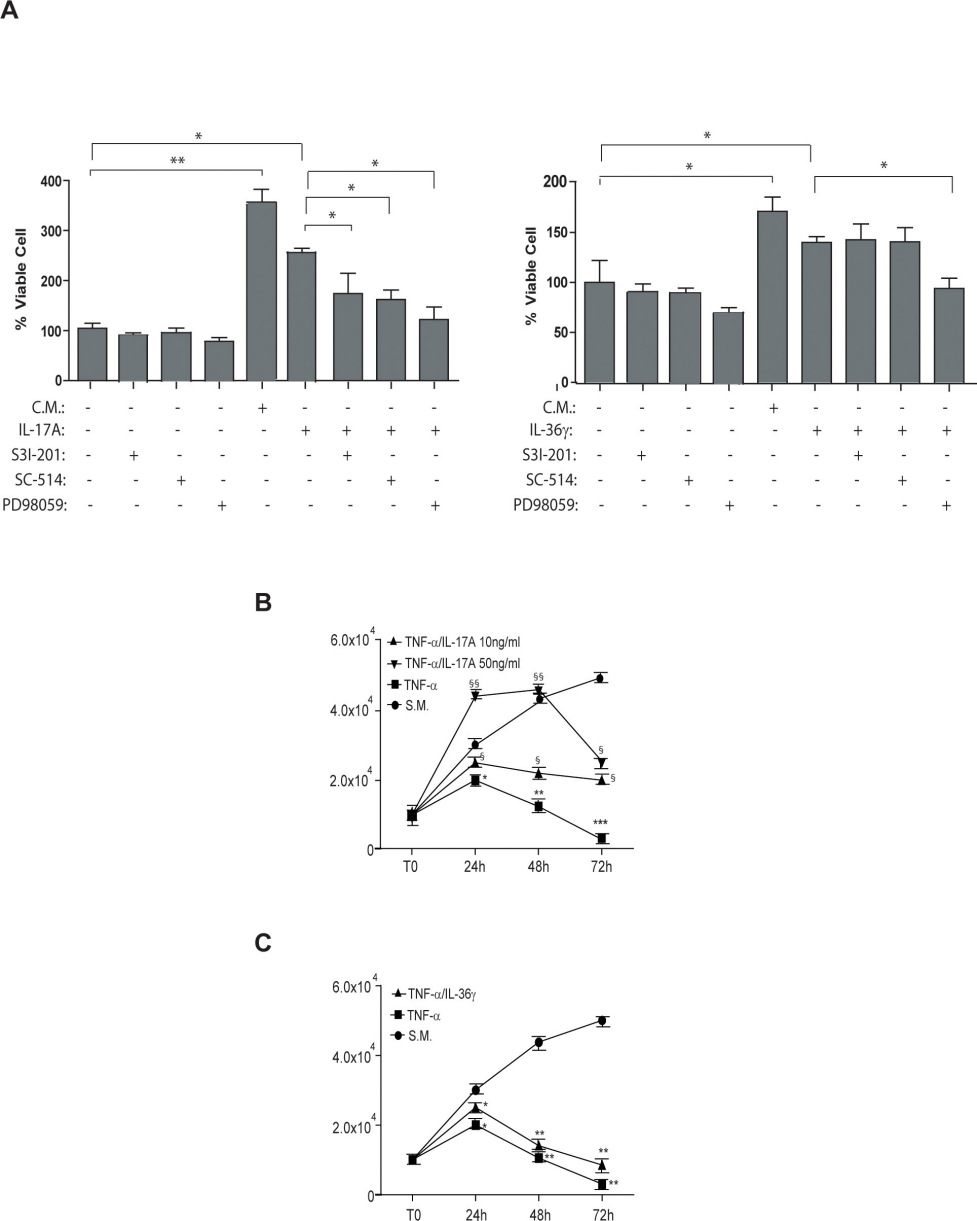
IL-17A and IL-36γ induce HDMEC proliferation through common and specific intracellular signaling pathways. **A.** CyQUANT proliferation assay was performed to determine HDMEC proliferation of cells grown in EGM as a complete medium (C.M.) or in EBM as a starvation medium (S.M.) in presence of IL-17A (50 ng/ml; left graph), IL-36γ (50 ng/ml; right graph) with addition of the S3I-201 STAT3 inhibitor, the SC-514 NF-κB inhibitor or the PD98059 ERK1/2 inhibitor for 48 hours. As controls, S3I-201, SC-514 or PD98059 were used alone in S.M. Data are shown as the percentage of mean values of fluorescence intensity obtained from three independent experiments ± SD. *p≤0.05, ** *p≤*0.01 calculated by One-way ANOVA comparing HDMEC grown in S.M. with HDMEC grown in C.M.; IL-17A- (left graph) or IL-36γ - treated cells (right graph) with untreated cells or with each inhibitor treatment as indicated by the connecting bars. **B.** HDMEC cells were grown in S.M. in the presence or absence of 10 ng/ml TNF-α, administered alone or in combination with 10 or 50 ng/ml IL-17A, for the indicated time points. **C.** HDMEC cells were grown in S.M. in the presence or absence of 10 ng/ml TNF-α, administered alone or in combination with 50 ng/ml IL-36γ, for the indicated time points. In **B** and **C**, proliferation was evaluated by cell counts using trypan blue exclusion test. Data are shown as mean values of viable cell counts obtained from three independent experiments ± SD as calculated by One-way ANOVA. In (B) *p≤0.05, **p≤0.01 comparing TNF-α-treated cells with S.M.; §*p*≤0.05, §§*p*≤0.01 comparing TNF-α/IL-17A 10 ng/ml and TNF-α/IL-17A 50 ng/ml with TNF-α-treated group; in (C) *p≤0.05, **p≤0.01 comparing TNF-α or TNF-α/ IL-36γ with S.M.

To analyze the involvement of STAT3, ERK1/2 or NF-κB in regulating HDMEC proliferation mediated by IL17-A or IL-36γ, proliferation was evaluated in the presence of STAT3 (S3I-201), NF-κB (SC-514) or ERK1/2 (PD98059) chemical inhibitors. As shown in Fig 2A, we found that inhibition of ERK1/2 significantly impaired IL17-A- and IL-36γ-induced HDMEC proliferation, whereas STAT3 and NF-κB inhibition only influenced HDMEC proliferation in response to IL-17A (Fig 2A). The three inhibitors used alone as a control did not significantly alter cell proliferation (Fig 2A).

Interestingly, when IL-17A was given in combination with TNF-α, it partially reverted the anti-proliferative effect of TNF-α within the 48 hours, at both 10 ng/ml and 50 ng/ml concentrations (Fig 2B). At 72 hours of treatment, despite of the reduction of the number of viable cells, the presence of IL-17A at both concentrations contributed to the survival of a higher number of HDMECs, when compared to HDMECs treated with TNF-α alone (Fig 2B). However, differently from IL-17A, IL-36γ had a limited potential in protecting cells from the anti-proliferative effect of TNF-α (Fig 2C).

**Fig 3:**
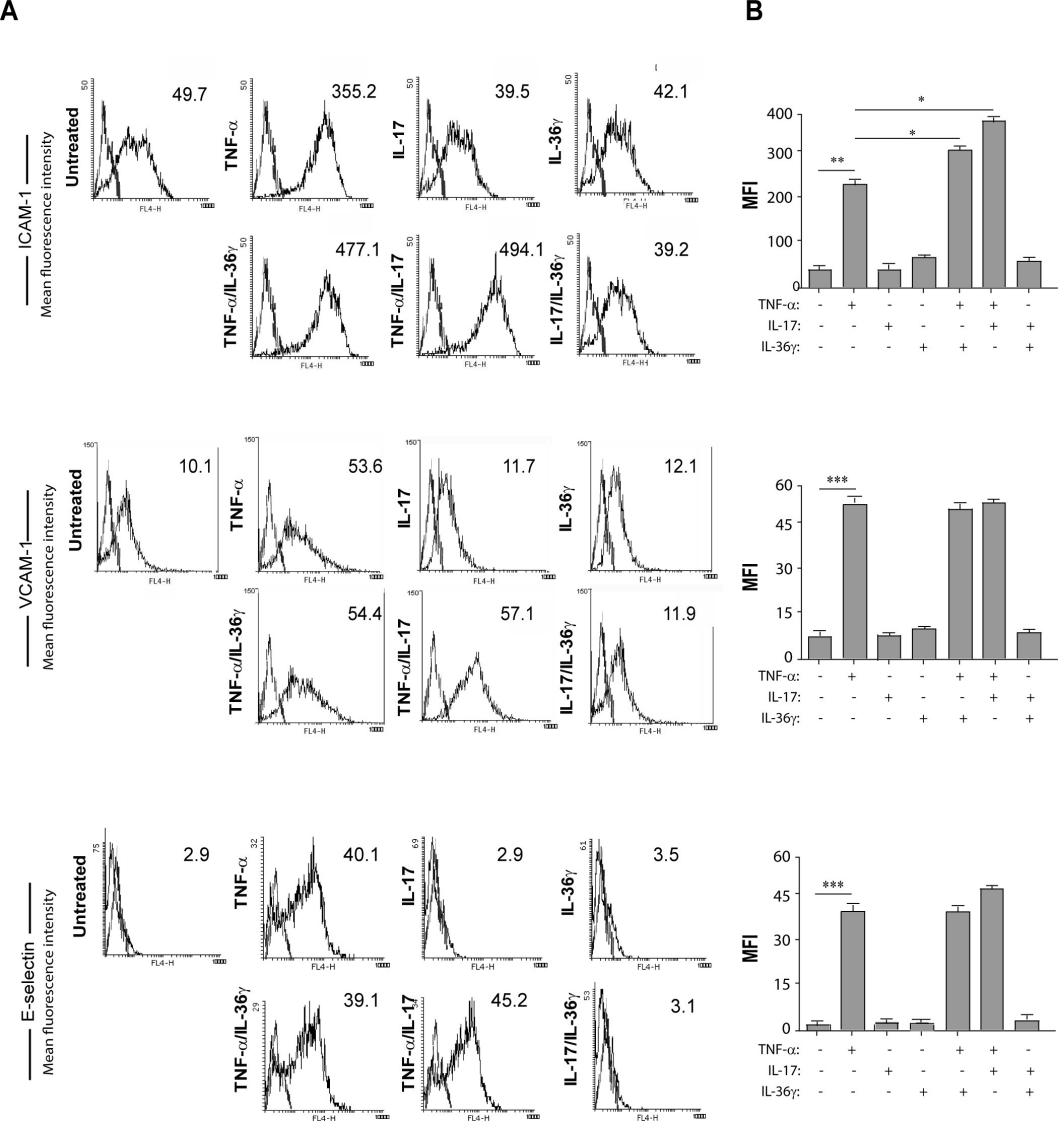
Both IL-17A and IL-36γ in combination with TNF-α increase ICAM-1 expression. **A.** Flow cytometry analysis of ICAM-1, VCAM-1 and E-Selectin expression was performed on HDMECs stimulated for 48 hours with IL-36γ (50 ng/ml), IL-17A (50 ng/ml) or TNF-α (10 ng/ml) alone or in different combinations, i.e. TNF-α/IL-36γ, TNF-α/IL-17A or IL-17A/IL-36γ. Data shown are expressed as mean fluorescence intensity (MFI) and represent one out of three independent experiments. B. Graphs show MFI values of three different experiments performed independently ± SD. *p≤0.05, **p≤0.01, ***p≤0.005, as calculated by Kruskal Wallis analysis, comparing TNF-α-treated group with S.M. and TNF-α/IL-36γ- or TNF-α/IL-17A- with TNF-α- treated group.

In inflammatory conditions, ECs up-regulate membrane receptors that are fundamental for leukocyte adhesion and extravasation from the blood flow into the inflamed tissue [31]. To study the expression of adhesion molecules on the HDMEC membrane following treatment with IL-17A or IL-36γ, flow cytometry analysis was performed on HDMECs stimulated for 48 hours with: i) IL-17A, administered alone or in combination with TNF-α; ii) IL-36γ, alone or in combination with TNF-α; iii) IL-17A in combination with IL-36γ. The lower concentration of IL-17A (10 ng/ml) was used in order to minimize the effect of IL-17A on cell proliferation and to put in evidence its inflammatory role. As shown in Fig 3, treatment of HDMECs with IL-17A or IL-36γ alone did not affect membrane expression of ICAM-1. In a similar way, HDMEC treatment with IL-36γ in combination with IL-17A did not influence membrane expression of ICAM-1. On the other hand, both IL-17A and IL-36γ significantly increased TNF-α-mediated induction of ICAM-1. No significant modulation of expression of either VCAM-1 or E-Selectin (Fig 3) could be observed after IL-17A or IL-36γ treatment, given alone or together with TNF-α.

To investigate the effects of IL-17A on HDMEC secretion of soluble inflammatory mediator, we used a Bio-Plex Pro^TM^ assay in which several inflammatory molecules could be simultaneously analyzed. As a comparison, HDMECs were treated with TNF-α alone or in combination with IL-17A. As shown in Table 1, IL-6, CXCL8/IL-8, G-CSF, CXCL10/IP-10 and CCL2/MCP-1 were significantly up-regulated following cell treatment with IL-17A alone, even if to a less extent compared to that observed with TNF-α treatment. Secretion of these five cytokines, together with IL-1RA, was significantly augmented upon stimulation with both IL-17A and TNF-α, with an additive effect in respect to TNF-α treatment alone. The complete Bio-Plex Pro^TM^ data are available as S1 Table.

**Table 1:** Bio-Plex 27-Plex cytokine analyses of supernatants from HDMECs treated with IL-17A alone or in combination with TNF-α.

Molecules	Untreated		TNF-α		IL-17A		TNF-α + IL-17A	
	Mean	SD	Mean	SD	Mean	SD	Mean	SD
IL-1RA	5.4	0.2	121.0^§^	7.1	18.0	1.5	196.1^#^	4.8
IL-6	72.3	13.0	5566.5^§^	506.0	1284.3^§^	147.3	13349.9^#^	475.5
IL-8	157.3	6.4	2507.7^§^	202.9	1093.9^§^	4.3	2727.2^#^	9.7
G-CSF	1.1	0.9	3580.6^§^	138.5	246.4^§^	27.4	86336.6^#^	579.7
GM-CSF	35.2	15.5	555.5^§^	18.7	56.5	5.8	1050.5^#^	127.2
IP-10	ND	ND	18567.4^§§^	807.8	29.5^§^	10.5	23777.9^#^	110.3
MCP-1	44.1	14.8	150.6^§^	40.7	146.6^§^	3.7	207.0	1.7
RANTES	2.8	0.5	2614.6^§§^	57.9	5.8	0.2	2796.4	68.4

Data are expressed as means of pg/ml ± SD obtained from duplicate samples. ND = not detected. *P* values were calculated by comparing: i) TNF-α- or IL-17A-treated *vs* untreated groups

^**§**^*p*<0.05 and

^**§§**^*p*<0.01; ii) TNF-α + IL-17A- *vs* TNF-α-treated groups

^#^*p<*0.05. Statistical significance was calculated by Mann-Whitney *U* test.

To validate the Luminex data, inflammatory molecule release by HDMEC was also evaluated by ELISA. In addition, supernatants from HDMEC treated with IL-36γ or IL-17A, alone or in combination with TNF-α, were tested for chemokine/cytokine content. Analysis of secreted proteins confirmed most of the results obtained by using Bio-Plex Pro^TM^ assay. As shown in Table 2, the release of G-CSF, CXCL10/IP-10, CXCL8/IL-8, CCL2/MCP-1, CCL5/RANTES and IL-6 was significantly augmented by single cytokine addition, with the exception of CCL2/MCP-1 and CCL5/RANTES secretion that was not regulated by IL-17A. TNF-α was confirmed to be the major inducer of the cytokines and chemokines analyzed. Moreover, secretion of G-CSF, CXCL10/IP-10, CXCL8/IL-8, CCL2/MCP-1 and IL-6 was significantly increased following HDMEC treatment with a combination of either IL-17A or IL-36γ and TNF-α., whereas CCL5/RANTES significantly augmented in supernatants of HDMECs treated with the combination of IL-36γ and TNF-α.

**Table 2:** ELISA confirmed the results obtained in the Luminex assay.

Cytokines and chemokines	Untreated	IL-17A	TNF-α	IL-36γ	IL-17A + TNF-α	IL-36γ + TNF-α
**G-CSF**	ND	0.072±0.006*	2.07±0.04*	0.22±0.0041*	30.14±3.50^**§§**^	14.94±2.89^**§§**^
**IP-10**	0.29±0.02	1.40±0.04*	102.1±2.3**	0.45±0.04	155.21±13.1^**§**^	196.56±14.3^**§**^
**IL-8**	9.0±2.4	29.6±3.2*	106.8±2.3**	23.2±3.6*	160.95±12.9^**§**^	167.10±13.5^**§**^
**MCP-1**	18.6±2.1	23.3±1.9	110.7±9.4**	48.94±2.4*	152.40±11.2^**§**^	160.41±14.3^**§**^
**RANTES**	ND	ND	30.6±2.1**	0.022±0.002*	42.55±1.4	45.49±2.8^**§**^
**IL-6**	0.35±0.01	2.89±0.02*	21.17±3.6**	0.61±0.02*	90.08±2.7^**§§**^	35.80±1.8^**§**^

Data are expressed as means of ng/10^6^ cells ± SD obtained from three independent experiments. P values are calculated by comparing: i) single cytokine-treated versus untreated groups

*p≤0.05 and

**p≤0.01; ii) IL-17A+TNF-α- or IL-36γ+TNF-α-treated versus TNF-α-treated groups

^§^*p*≤0.05 and

^§§^*p*≤0.01. Statistical significance was calculated by Mann-Whitney U test.

**Fig 4:**
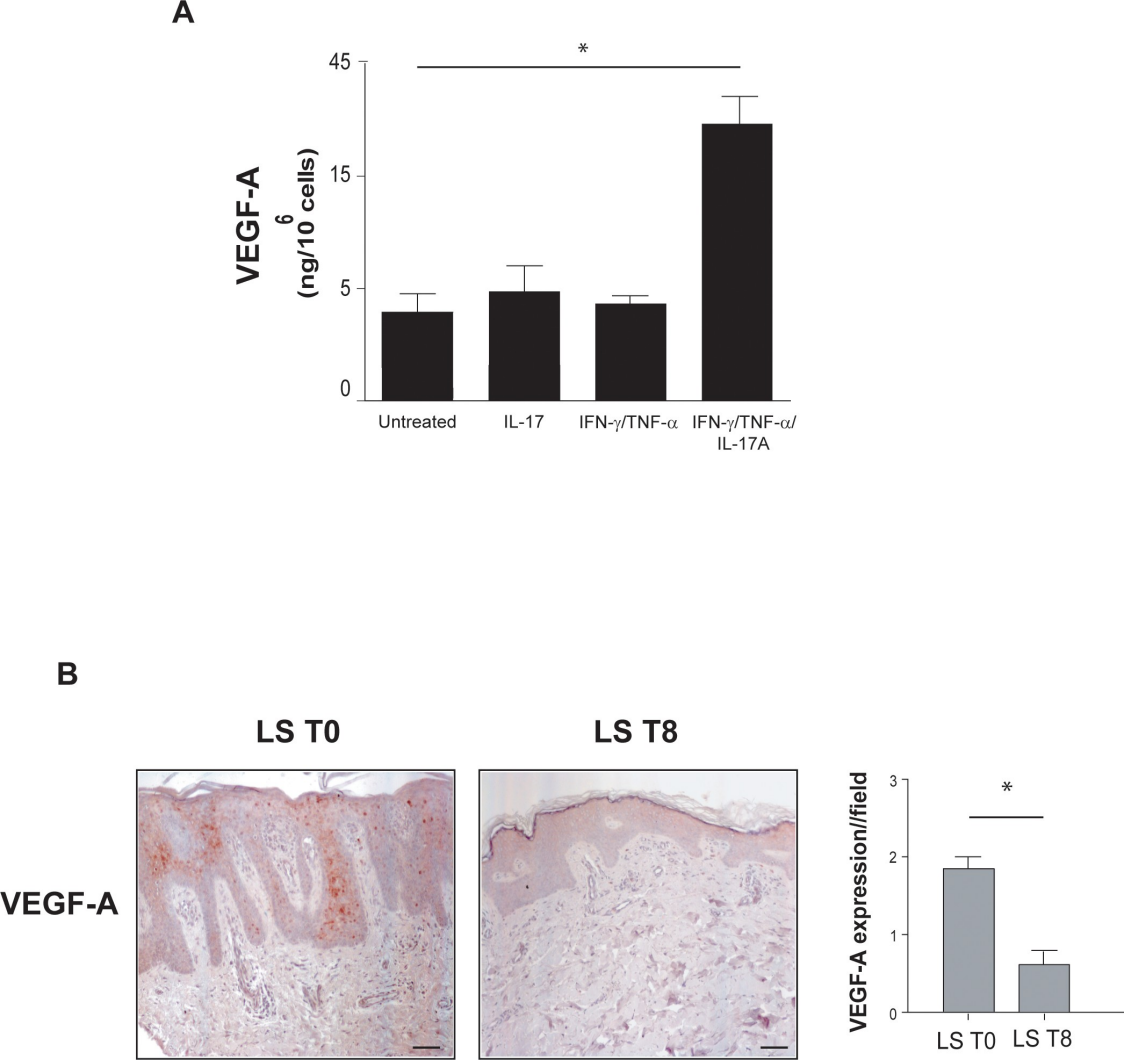
IL-17A in combination with TNF-α induces VEGF-A secretion both *in vitro* and *in vivo*. **A.** Supernatants from three different human keratinocyte strains were analysed for VEGF-A secretion by ELISA after treatment with IL-17A, alone or in combination with TNF-α and IFN-γ. Results are presented as the mean of ng/10^6^ cells ± SD of the values obtained from the different strains in three independent experiments, *p≤0.05 in respect to untreated cells as assessed by Student’s *t* test. **B.** VEGF-A immunohistochemical staining (red) of patients' lesional psoriatic skin (LS), before (T0, i) and after an eight-week treatment with Secukinumab (T8, ii). Representative sections of skin specimens from three patients are shown (bars = 100 μm). Graphs show the mean values ± SD of four-stage score values for VEGF-A expression in three different fields. *p ≤ 0.05, as assessed by Mann–Whitney *U* test.

The pro-angiogenic role of IL-17A has been often ascribed to its ability to stimulate skin keratinocytes to release angiogenic factors, especially VEGF-A [32,33]. Thus, we analyzed VEGF-A protein secretion by human keratinocytes treated or not with IL-17A and combination of IL-17A, IFN-γand TNF-α. As shown in Fig 4A, IL-17A alone does not significantly induce the secretion of this angiogenic growth factor but it strongly synergizes with IFN-γ and TNF-α in stimulating VEGF-A release by human keratinocytes.

There is evidence that VEGF-A is a primary angiogenic factor in psoriasis [34]. Serum levels of VEGF-A are higher in patients affected by psoriasis than in healthy controls, correlate with the Psoriasis Area and Severity Index (PASI) and diminish after treatment with psoralen plus ultraviolet-A (PUVA) or acitretin [35]. However, limited data are available about VEGF-A lowering after treatment of patients with anti-IL-17 antibodies. Thus, we analyzed VEGF-A expression in skin biopsies from plaque lesions of patients undergone anti-IL-17A Secukinumab therapy. As shown in Fig 4B, VEGF-A was strongly expressed in the suprabasal keratinocyte layer of the lesional skin and it was reduced after an 8-week Secukinumab treatment (Fig 4B).

**Fig 5:**
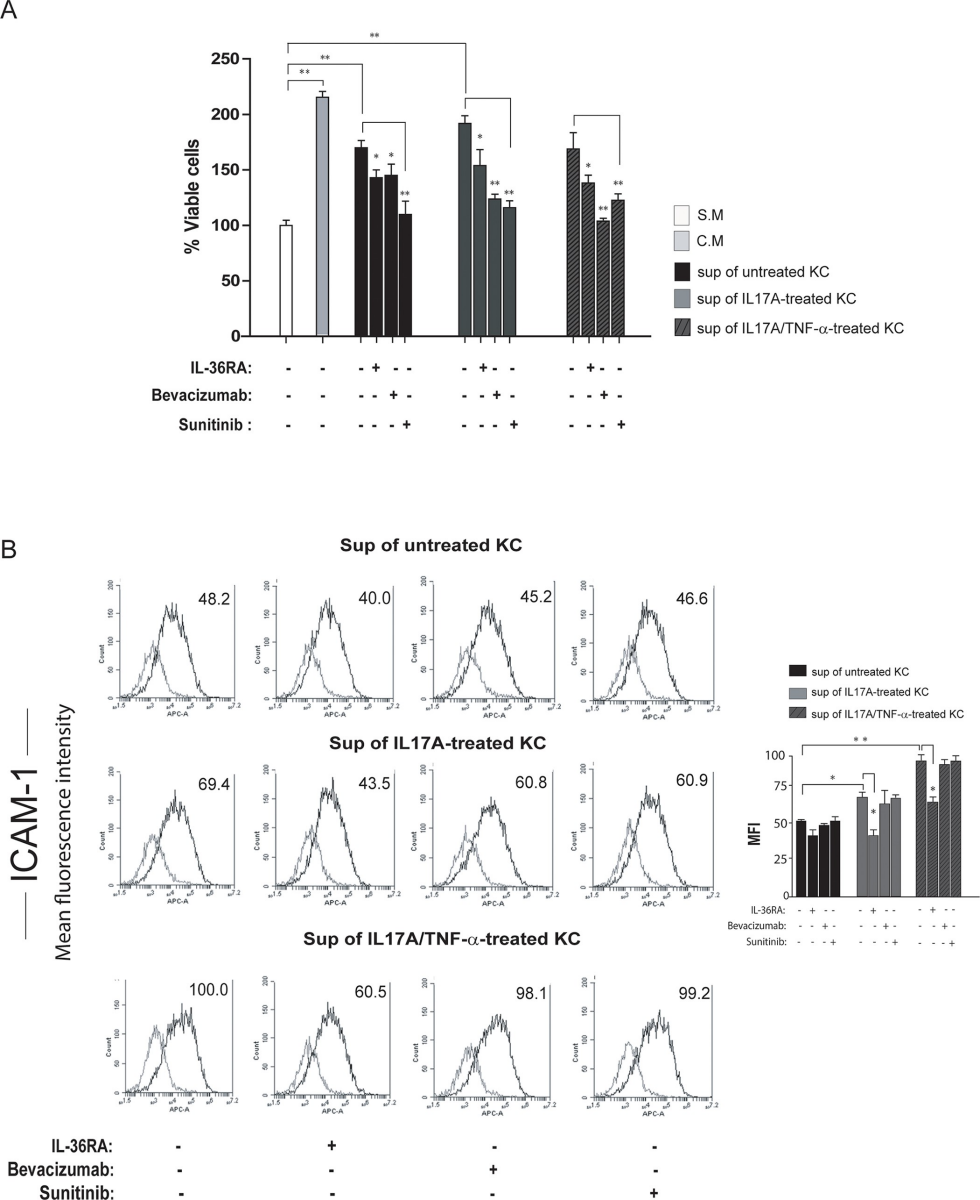
Crosstalk between keratinocytes and HDMECs is mediated by both VEGF-A and IL-36γ. **A**. Cell proliferation was evaluated by CyQUANT assay performed on HDMECs in EBM as a starving medium (S.M.), EGM as a complete medium (C.M.) or stimulated for 24 and 48 hours with the supernatants of untreated, IL-17A- or IL-17A/TNF-α-treated keratinocytes (KC), in the presence or not of IL-36RA, Bevacizumab or Sunitinib. Data are shown as the percentage of mean values of fluorescence intensity obtained from three independent experiments ± SD. *p≤0.05, ** p≤0.01 by Kruskal Wallis test. **B.** ICAM-1 expression was evaluated by flow cytometry analysis on HDMECs stimulated for 36 hours with supernatants (Sup) of untreated, IL-17A- or IL-17A/TNF-α-treated KC in the presence or not of IL-36RA, Bevacizumab or Sunitinib. Data are expressed as mean fluorescence intensity (MFI) and represent one out of three independent experiments. Graphs show the MFI mean values of three different experiments performed ± SD. *p≤0.05, **p≤0.01 by Kruskal Wallis test.

To clarify the role of VEGF-A in the IL-17/IL-36 axis and in the crosstalk between skin keratinocytes and HDMECs, we incubated HDMECs with culture medium conditioned by keratinocytes treated with IL-17A alone or in combination with TNF-α and analyzed proliferation and adhesion molecule expression. In parallel, we treated HDMECs with the IL-36 receptor antagonist (IL-36RA), with the tyrosine kinase inhibitor Sunitinib, that blocks the activity of either VEGFs or platelet-derived growth factor (PDGF) receptors as well as the signaling associated to CD117/c-kit [36] or with the monoclonal antibody Bevacizumab that interferes with VEGF-A binding to VEGFR-2 [37]. As shown in Fig 5A, stimulation with supernatants of untreated keratinocyte induced HDMEC proliferation compared to the control, and cell proliferation was significantly reduced by either IL-36RA, Sunitinib or Bevacizumab. These results fit with the similar secreted amounts of IL-36γ and VEGF-A observed in the conditioned medium of untreated keratinocytes (S1C Fig and Fig 4A) and thus with the angiogenic effect of these cytokines on HDMECs. Importantly, HDMEC treatment with the supernatant of IL-17A-stimulated keratinocytes further increased cell proliferation. This increment was also blocked by either IL-36RA, Sunitinib or Bevacizumab (Fig 5A). Unexpectedly, stimulation with both IL-17A and TNF-α-treated keratinocyte supernatants did not further induce HDMEC proliferation compared to untreated keratinocyte conditioned medium, but inhibition of either VEGF-A or IL-36γ was again effective in reducing cell proliferation (Fig 5A). In parallel to proliferation studies, FACS analyses of HDMECs treated with keratinocyte-conditioned media showed that supernatants of IL-17A- or IL-17A/TNF-α-treated keratinocytes significantly up-regulated ICAM-1 expression by HDMECs, as compared to treatments with supernatant of untreated keratinocytes (Fig 5B). Of note, only IL-36RA treatment significantly reduced ICAM-1 expression on HDMECs induced by supernatants conditioned by IL-17A- and IL-17A/TNF-α-treated keratinocytes (Fig 5B). In fact, Sunitinib or Bevacizumab did not alter ICAM-1 expression in all experimental conditions, indicating that keratinocyte-derived VEGF-A had not an active role in regulating ICAM-1 expression on HDMECs (Fig 5B).

**Fig 6:**
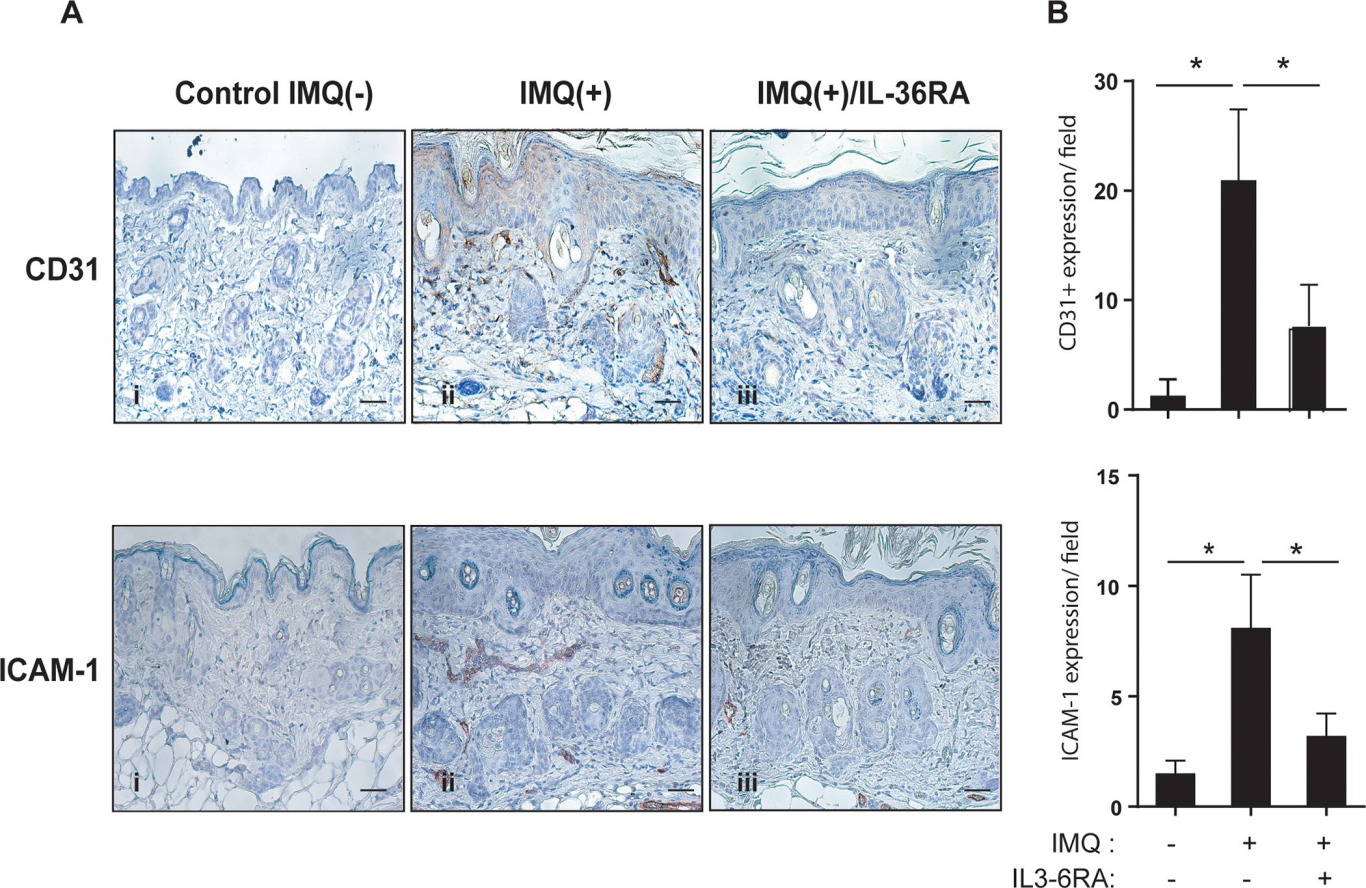
IL-36R inhibition reduces CD31 and ICAM-1 expression by HDMEC in the IMQ-induced psoriasis-like mouse model. **A.** Immunohistochemical analyses of CD31 and ICAM-1 performed on sections of mouse skin obtained from untreated [Control IMQ(-)], IMQ-treated [IMQ(+)], and IMQ-treated mice subjected to IL-36RA administration [IMQ(+)/IL-36RA]. Bars = 200 μM. Graphs show the mean number of CD31- or ICAM-1-positive cells ± SD. *p≤0.05 by Mann-Whitney *U* test. Significance were calculated comparing Control IMQ(-) with IMQ(+) group, and IMQ(+) with IMQ(+)/IL-36RA group.

We further investigated the effects of IL-36R inactivation on induction of ICAM-1 by HDMEC using an IMQ-induced psoriasiform dermatitis mouse model. Daily application of IMQ on mouse back skin induced inflamed scaly skin lesions resembling plaque psoriasis, characterized by increased epidermal proliferation, accumulation of immune cell infiltrate, neo-angiogenesis and high expression of IL-23, IL-17A and IL-36 [38,39]. As shown in Fig 6, immunohistochemical analysis indicated increased expression of CD31 and ICAM-1 in microvascular endothelial cells of the IMQ-treated mice in comparison to control mice. Subcutaneous injection of IL-36RA for 5 days, concomitantly to topical IMQ application, induced a strong decrease of CD31-positive endothelial cells (60% reduction). Consistently with *in vitro* results, administration of IL-36RA also reduced the number of ICAM-1-positive endothelial cells (30% decrease).

## Discussion

It is well known that both IL-17A and IL-36γ activate pathogenic pathways in different cell types in psoriatic skin, especially in resident skin cells such as keratinocytes and dermal EC. While much is known about the direct impact of IL-17A and IL-36γ on these skin cell types, no studies about their indirect inflammatory effects on EC mediated by keratinocytes have been reported so far. In this work, we highlight the strong IL-17A- and IL-36γ-dependent interplay between keratinocytes and ECs in psoriatic condition that leads to the establishment of a cytokine network responsible for the development and maintenance of the inflamed state. We clarify the action of IL-17A in the crosstalk between skin keratinocytes and ECs by investigating the involvement of IL-36γ and VEGF-A, both abundantly and constitutively produced by keratinocytes and augmented after cytokine stimulation. In fact, we showed that IL-17A promoted EC proliferation *in vitro* either directly or indirectly through induction of IL-36γ release by keratinocytes. Moreover, IL-17A acted together with TNF-α in inducing both IL-36γ and VEGF-A in human keratinocytes. Consistently, when HDMECs were stimulated with supernatants of IL-17A- or IL-36γ/TNF-α-treated keratinocytes, both IL-36γ and VEGF-A were found to be responsible for the observed increase of HDMEC proliferation, as demonstrated by inhibiting IL-36γ or VEGF-A with IL-36RA or with Sunitinib or Bevacizumab, respectively. Our results support the idea that IL-36 cytokines and particularly IL-36γ are important angiogenic mediators of IL-17A action. Interestingly, treatment with supernatants of IL-17A- and TNF-α-treated keratinocytes did not further enhance HDMEC proliferation in respect to stimulation with supernatants of untreated keratinocytes. Therefore, we can speculate that the IL-17A/TNF-α combination induces the release by keratinocytes of additional mediators able to counteract the proliferative action of IL-36γ and/or VEGF-A on HDMECs.

Concerning the angiogenic role of IL-17A in psoriasis, it is important to emphasize the direct effect of this cytokine on the proliferation of IL-17R-bearing ECs, as previously reported [14,17] and confirmed in this study. At the molecular level, we observed that IL-17A supported EC proliferation by inducing activation of either NF-κB-, ERK1/2- or STAT3-dependent pathways, all known to be implicated in proliferation and survival processes of several cell types. IL-17A induced a rapid and sustained activation of NF-κB, as well as a biphasic activation of the ERK1/2 pathway, known to be intimately involved in the regulation of cell-cycle progression and cell proliferation [40]. IL-17A also mediated a late activation of STAT3, possibly having an indirect effect on STAT3-mediated molecular pathways. It is worth of noting that IL-17A could control EC growth indirectly by inducing IL-6 and downstream STAT3-dependent pathways [41]. Therefore, STAT3 activation by IL-17A could be indirect and depend on IL-17A-induced IL-6 and its autocrine action on ECs, thus ultimately up-regulating pro-survival and pro-angiogenic genes in these cells. IL-36γ also induced EC proliferation, even though at a lower extent than IL-17A and by primarily activating ERK1/2 signaling. The stronger activation of ERK1/2 and, specifically, of the STAT3 pathway by IL-17A could explain the more pronounced mitogenic effect of IL-17A compared to that promoted by IL-36γ [27,42,43]. Furthermore, IL-36γ also induced activation of NF-κB, whose inhibition did not affect EC proliferation, thus functioning mainly as a pro-inflammatory mediator, as extensively described [29,44]. Other than having a mitogenic effect, we demonstrated that IL-17A counteracted the anti-proliferative effect of TNF-α, potentially activating the pro-survival ERK1/2 pathway. Indeed, IL-17A could protect keratinocytes from the pro-apoptotic effect of TNF-α, but a more detailed analysis of the molecular mechanisms underlying this process should be performed. A stronger activation of ERK1/2-dependent pathway by IL-17A could also explain why IL-36γ did not counteract the anti-proliferative effects of TNF-α as efficiently as IL-17A.

Our analysis of EC inflammatory responses showed that IL-17A or IL-36γ administered alone did not regulate the expression of ICAM-1, whereas together with TNF-α both induced this adhesion molecule in HDMECs. Importantly, we found that ICAM-1 expression by HDMECs could be also efficiently induced by supernatants from IL-17-treated keratinocytes in an IL-36-dependent manner, as demonstrated by blocking IL-36 cytokine action with IL-36RA. However, considering that IL-36γ alone does not directly induce ICAM-1 expression by HDMECs, it is possible that other IL-36 isoforms, such as IL-36β, could be responsible for the observed ICAM-1 expression. A role of IL-36 cytokines in adhesion molecule expression by ECs has been also proven *in vivo* using the IMQ-induced psoriasis-like mouse model. In the presence of an IL-36RA inhibitor, both ICAM-1 and CD31 expression were greatly reduced. This is the first demonstration that IL-36 cytokines contribute to the psoriatic phenotype of the IMQ-treated mice also influencing proliferation and inflammatory responses of ECs, other than regulating immune functions and differentiation of the epidermal compartment, as well as modulating immune cell recruitment and activation [29]. Of note, blocking of VEGF-A in keratinocyte-derived supernatants by Sunitinib or Bevacizumab treatment did not alter membrane expression of ICAM-1. These data are in line with previous findings demonstrating that VEGF-A inhibitors do not down-regulate ICAM-1, but could even induce ICAM-1 up-regulation [45]. Induction of ICAM-1 expression by VEGF-A inhibition is an emerging aspect that supports the association of immunotherapy and anti-angiogenic therapy in cancer treatment. In fact, blocking angiogenesis could make the tumor more accessible and vulnerable to the immune system [46]. A recent paper by van Hooren and colleagues [47] demonstrated the up-regulation of ICAM-1 expression on tumor ECs treated with Sunitinib and an agonistic anti-CD40 monoclonal antibody. CD40 is a member of the TNF super family and, in immune cells, its expression is induced by several pro-inflammatory cytokines such as IL-36γ itself. Nevertheless, in a psoriasis microenvironment, the lack of an anti-inflammatory effect of VEGF inhibition could be responsible for the limited results of anti-angiogenic treatment of this pathology [48].

Despite what previously reported [11], we clearly demonstrate that IL-17A alone has a direct pro-inflammatory effect on ECs, by inducing cytokines and chemokines, such as IL-6, CXCL8/IL-8, G-CSF, CXCL10/IP-10 and CCL2/MCP-1. In addition, IL-17A assisted or synergized with TNF-α on ECs, as indicated in other studies [13], either through induction of membrane proteins, such as ICAM-1, or by secretion of inflammatory soluble mediators.

Our data support the hypothesis that targeting IL-17A should result in an improvement of the EC damage observed in psoriasis patients. This chronic microvascular damaging would lead through time to the cardiovascular co-morbidities recurrently associated to psoriasis. Clinical trials indicate that treatment with biological drugs, such as Secukinumab, that target the IL-17A signaling pathway, markedly improves disease outcome. These IL-17-targeting drugs are generally well tolerated and constitute a good alternative to other biological compounds that target TNF-α. Anti-TNF-α treatment with Infliximab of psoriasis patients significantly reduce the levels of VEGF-A [49]. Similar data are not fully available for the IL-17-targeting compounds. As for Secukinumab, the 52-week, randomized, double-blind, placebo-controlled, exploratory trial CARIMA showed a tendency of psoriasis patients in ameliorating endothelial functions measured by flow-mediated dilation [50]. On the other hand, in mouse models of psoriasis, vascular inflammation, evaluated through the measure of circulating inflammatory cytokines and chemokines, and vascular dysfunction, analyzed *ex-vivo* by vascular responsiveness to vasodilators, were correlated with the severity of skin lesions and levels of IL-17A. In a model of moderate to severe form of psoriasis with a late onset, anti-IL-17A treatment have beneficial effects on both vascular inflammation and dysfunction [51]. Our results are consisting with these findings and further indicate that Secukinumab treatment reduces VEGF-A presence in the psoriatic skin. In the future, for a better management of psoriasis cardiovascular co-morbidities, the association of anti-IL-17A therapy with an anti-IL-36γ treatment should be considered.

## Supporting information

S1 Fig HDMEC express IL-17 and IL-36 receptors and secrete the three IL-36 isoforms. **A.** Western blotting analysis confirmed expression by HDMEC of both IL-17RA and IL-1Rrp2. Treatment with either IL-17A, IL-36γ, TNF-α or combination of the cytokines (TNF-α+IL-17A; TNF-α+ IL-36γ) did not induce receptor expression by HDMEC. **B-C.** ELISA assays with supernatant from HDMEC (B) and from keratinocytes (KC) (C) for the three isoforms of IL-36. Treatment with IL-17A or TNF-α, or combination of IL-17A and TNF-α in HDMEC or IFN-γ, TNF-α and IL-17A in keratinocytes significantly augmented protein secretion. Results are presented as the mean (pg or ng/10^6^ cells ± SD) from independent experiments; p*≤0.05; **≤0.01 compared with untreated cells by Student´s t test.
(TIF)

S2 Fig IL-17A and IL-36γ induce HDMEC proliferation. **A.** HDMEC cells grown in EGM as a complete medium (C.M.) or in EBM as a starvation medium (S.M.) in the presence or absence of 10 or 50 ng/ml IL-17A, for the indicated time points. **B.** HDMECs were treated with IL-36γ, alone or in combination with IL-17A in S.M. or left untreated. Proliferation was evaluated by cell counts using trypan blue exclusion test. Data are shown as mean values of viable cell counts obtained from three independent experiments ± SD. *p≤0.05, ** *p≤*0.01 as calculated by One-way ANOVA comparing each experimental condition with S.M.
(TIF)

S1 Table Bio-Plex 27-Plex cytokine analyses of supernatants from HDMECs treated with IL-17A alone or in combination with TNF-α. *P* values were obtained by Mann-Whitney *U* test analysis.
(PDF)

S1 Raw images (PDF)
