# Supplementary figures and images for: Interleukin (IL)-17/IL-36 axis participates to the crosstalk between endothelial cells and keratinocytes during inflammatory skin responses
Source: PLoS One. 2020 Apr 30;15(4):e0222969. doi: 10.1371/journal.pone.0222969 (PMC7192413; doi:10.1371/journal.pone.0222969)

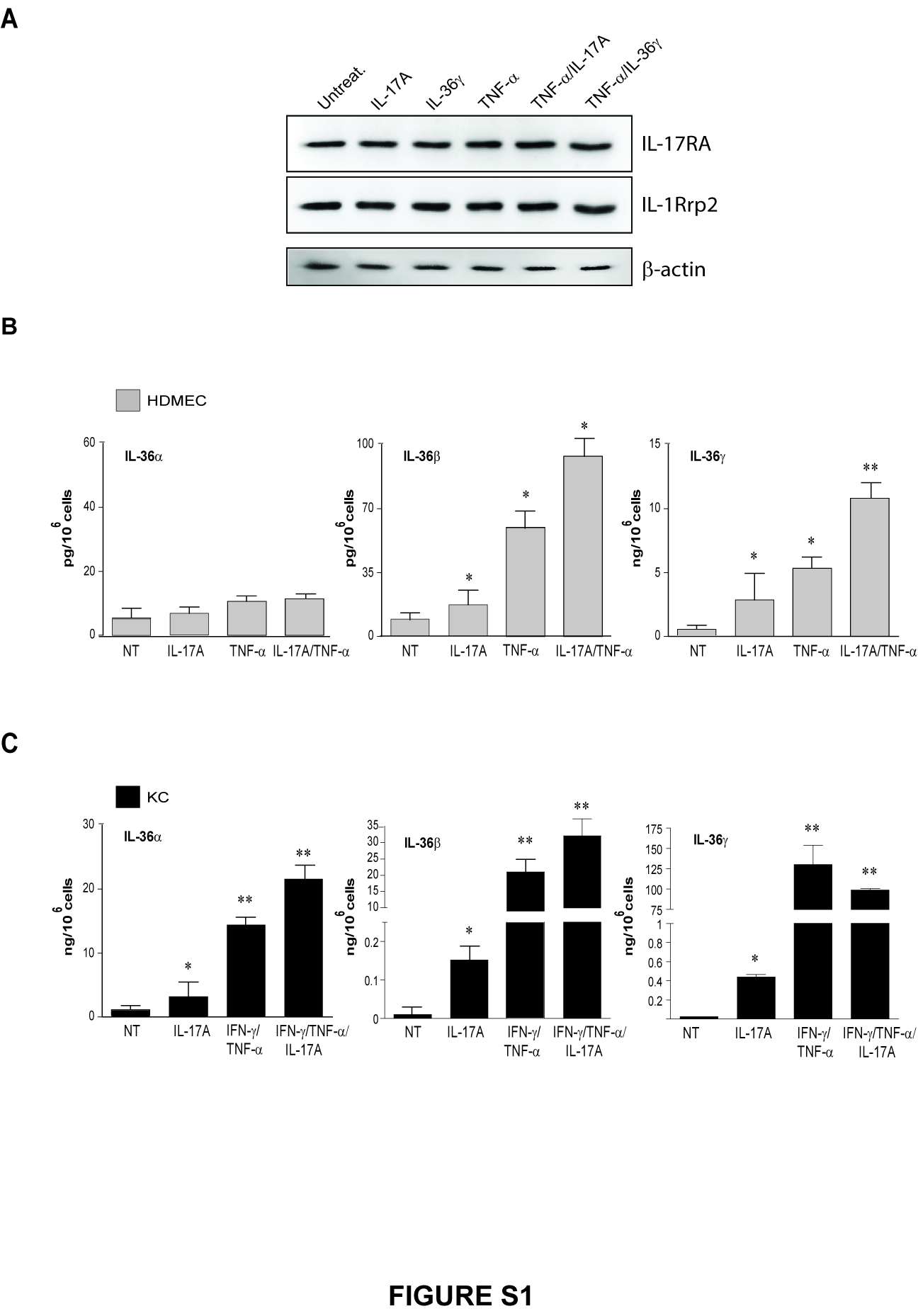

Supplement: S1 Fig — A. Western blotting analysis confirmed expression by HDMEC of both IL-17RA and IL-1Rrp2. Treatment with either IL-17A, IL-36γ, TNF-α or combination of the cytokines (TNF-α+IL-17A; TNF-α+ IL-36γ) did not induce receptor expression by HDMEC. B-C. ELISA assays with supernatant from HDMEC (B) and from keratinocytes (KC) (C) for the three isoforms of IL-36. Treatment with IL-17A or TNF-α, or combination of IL-17A and TNF-α in HDMEC or IFN-γ, TNF-α and IL-17A in keratinocytes significantly augmented protein secretion. Results are presented as the mean (pg or ng/106 cells ± SD) from independent experiments; p*≤0.05; **≤0.01 compared with untreated cells by Student´s t test. (TIF) [file pone.0222969.s001.tif]

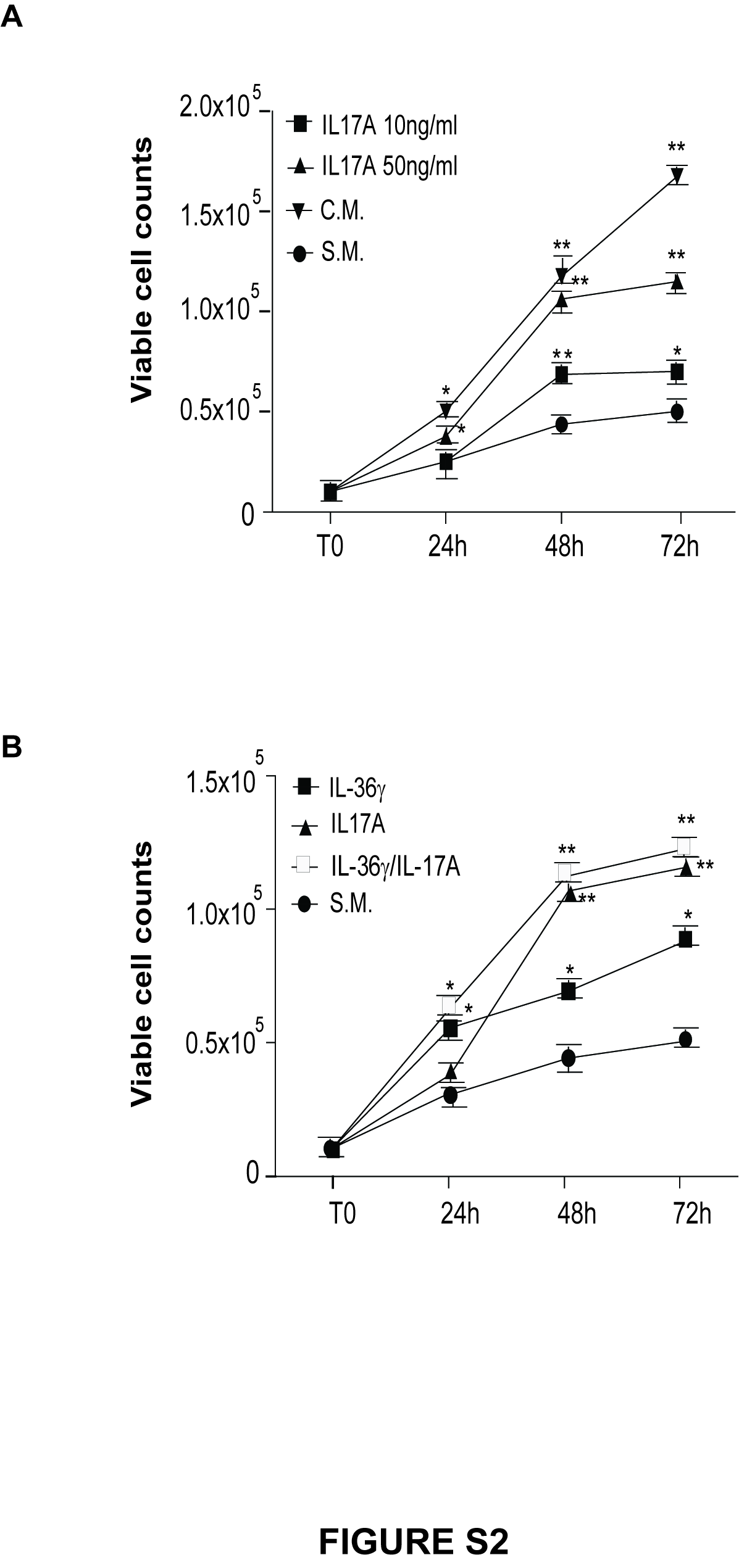

Supplement: S2 Fig — A. HDMEC cells grown in EGM as a complete medium (C.M.) or in EBM as a starvation medium (S.M.) in the presence or absence of 10 or 50 ng/ml IL-17A, for the indicated time points. B. HDMECs were treated with IL-36γ, alone or in combination with IL-17A in S.M. or left untreated. Proliferation was evaluated by cell counts using trypan blue exclusion test. Data are shown as mean values of viable cell counts obtained from three independent experiments ± SD. *p≤0.05, ** p≤0.01 as calculated by One-way ANOVA comparing each experimental condition with S.M. (TIF) [file pone.0222969.s002.tif]
