# Supplementary material for: Interleukin (IL)-17/IL-36 axis participates to the crosstalk between endothelial cells and keratinocytes during inflammatory skin responses
Source: PLoS One. 2020 Apr 30;15(4):e0222969. doi: 10.1371/journal.pone.0222969 (PMC7192413; doi:10.1371/journal.pone.0222969)
Supplement: S1 Table — P values were obtained by Mann-Whitney U test analysis. (PDF) [file pone.0222969.s003.pdf]

	A		B		C		D					
**Molecule**	**Untreated**		**IL-17A**		**TNFα**		**IL-17A +TNFα**		**P value B/A**	**P value C/A**	**P value D/A**	**P value D/C**
	**Mean**	**SD**	**Mean**	**SD**	**Mean**	**SD**	**Mean**	**SD**				
**IL-1b**	0.24	0.08	1.26	0.04	5.8	0.05	10.12	0.88	0.004	0.002	0.0042	0.0203
**IL-1ra**	5.43	0.25	18.13	1.52	120.99	7.11	196.09	4.78	0.073	0.0019	0.0003	0.065
**IL-2**	0.99	0.16	2.12	0.25	5.95	0.61	9.19	1.17	0.0332	0.079	0.01	0.073
**IL-4**	0.22	0.05	0.64	0.03	3.22	0.16	5.39	0.06	0.0093	0.0016	0.001	0.0031
**IL-5**	0.14	0.01	0.18	0.01	0.27	0.01	0.24	0.01	0.0194	0.0068	0.0111	0.1679
**IL-6**	72.29	13.07	1284.32	147.34	5566.55	506.01	13349.87	475.48	0.0073	0.0042	0.0006	0.004
**IL-7**	0.19	0.04	0.32	0.06	0.45	0.03	0.4	0.04	0.1178	0.0152	0.0344	0.2999
**IL-8**	157.32	6.44	1093.91	4.35	2507.7	202.92	2727.24	9.71	0.0001	0.0037	0.0001	0.2731
**IL-9**	0.57	0.13	47.5	0.33	1.86	0.18	37.77	19.52	0.0691	0.0601	0.0012	0.3101
**IL-10**	0.72	0.18	1.34	0.03	2.16	0.32	2.46	0.21	0.1102	0.1375	0.1071	0.7205
**IL-12(p70)**	0.56	0.07	1.49	0.36	2.08	0.55	2.62	0.07	0.0399	0.0422	0.0007	0.3101
**IL-13**	0.24	0.05	0.51	0.06	0.45	0.11	0.52	0.02	0.0015	0.0017	0.0014	0.5149
**IL-15**	4.65	0.22	9.9	0.73	17.62	1.34	17.4	0.67	0.0018	0.0056	0.0029	0.8519
**IL-17**	2.28	0.93	7789.43	711.2	16.56	0.42	9201.74	563.36	0.0253	0.1547	0.7319	0.0019
**Eotaxin**	ND	ND	6.02	0.21	10.03	3.01	15.26	1.27				0.151
**FGF basic**	16,00	2.28	35.75	1.36	108,00	15.73	97.5	1.63	0.011	0.0008	0.2325	0.0236
**G-CSF**	1.08	0.88	246.42	27.43	3580.61	138.49	86336.56	579.69	0.0001	0.0001	0.0001	0.0001
**GM-CSF**	35.25	15.54	56.46	5.83	555.51	18.72	1050.46	127.22	0.0573	0.0576	0.1157	0.1561
**IFN-g**	2.44	0.86	11.28	0.59	61.44	5.38	92.85	0.3	0.0015	0.0017	0.0014	0.5149
**IP-10**	ND	ND	29.48	10.48	18567.4	807.84	23777.91	110.34	0.1607	0.1266	0.1326	0.1717
**MCP-1(MCAF)**	44.11	14.88	146.61	3.69	150.62	40.71	207.01	1.7	0.1743	0.0437	0.0001	0.0148
**MIP-1a**	0.68	0.02	1.01	0.07	1.61	0.06	2.08	0.01	0.014	0.114	0.15105	0.1701
**PDGF-bb**	2.66	1.01	21.53	5.51	81.1	17.08	75.88	13.84	0.1959	0.1374	0.1343	0.1731
**MIP-1b**	1.05	0.01	3.62	0.32	3,00	0.01	5.96	0.23	0.1317	0.1232	0.1329	0.1723
**RANTES**	2.79	0.53	5.84	0.22	2614.62	57.98	2796.47	68.39	0.1981	0.6244	0.0163	0.0031
**TNF-a**	1.67	0.18	4.95	0.17	23620.2	8621.66	40655.45	6674.68	0.0023	0.0002	0.0003	0.103
**VEGF**	1.38	0.26	4.26	0.26	6.94	0.5	9.7	1.43	0.0038	0.0546	0.01229	0.1705
