# Supplementary material for: Interleukin (IL)-17/IL-36 axis participates to the crosstalk between endothelial cells and keratinocytes during inflammatory skin responses
Source: PLoS One. 2020 Apr 30;15(4):e0222969. doi: 10.1371/journal.pone.0222969 (PMC7192413; doi:10.1371/journal.pone.0222969)
Supplement: S1 Raw images — (PDF) [file pone.0222969.s004.pdf]

Blots used for Figure 1:

Filters were developed using the ECL-plus detection system (Amersham, Dubendorf, Switzerland), or for p-P65 detection, the SuperSignal West Femto kit (Pierce, Rockford, IL, USA) by using X-ray films in dark room.
Films, once developed, were then scanned with a standard computer scanner at 300dpi resolution.

Figure 1 Panel A: HDMEC stimulated or not with 50ng/ml of IL-17A at different time-points as described on the blots:

p-STAT3 and STAT3
molecular weight 86-88 kDa

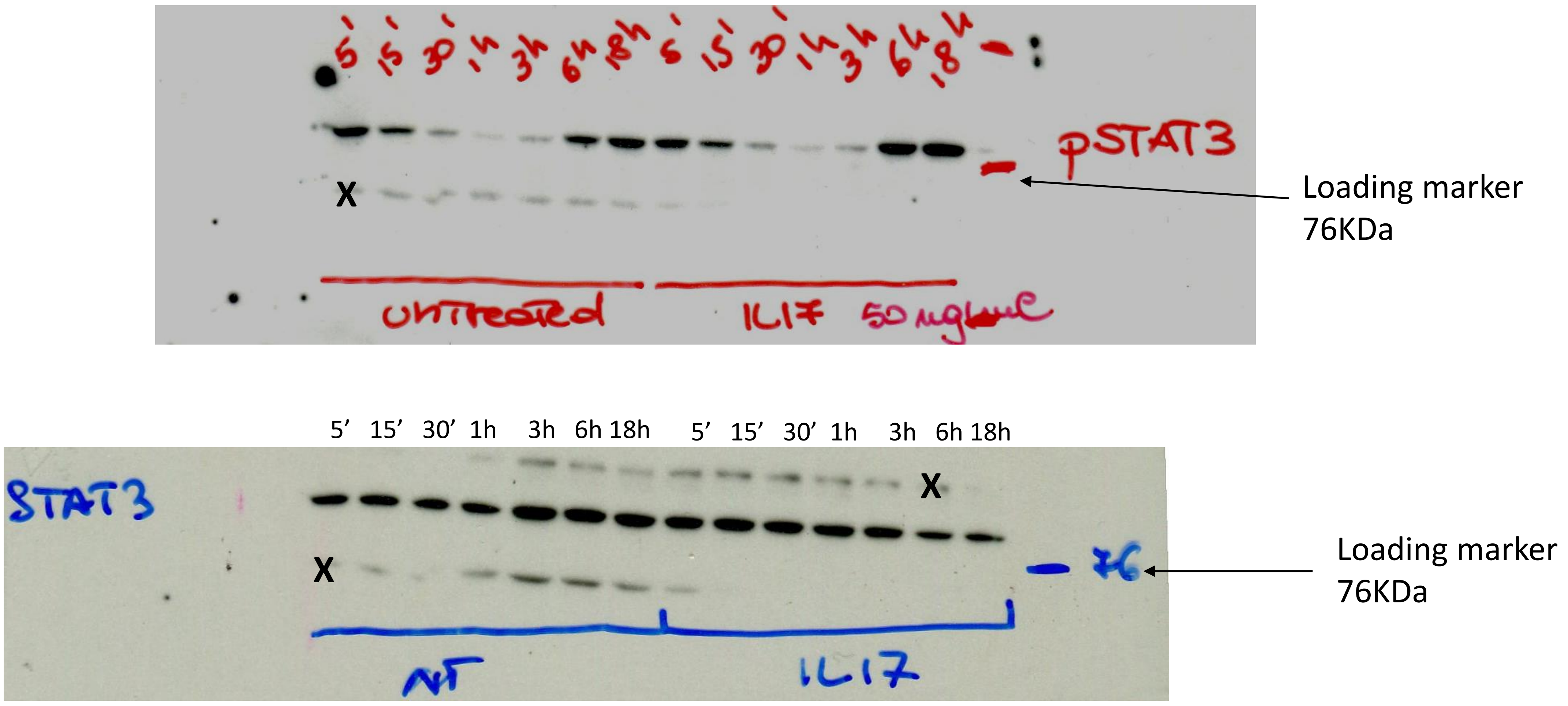

p-ERK and ERK
molecular weight: 42-44 kDa

Loading marker
50 kDa and38 kDa

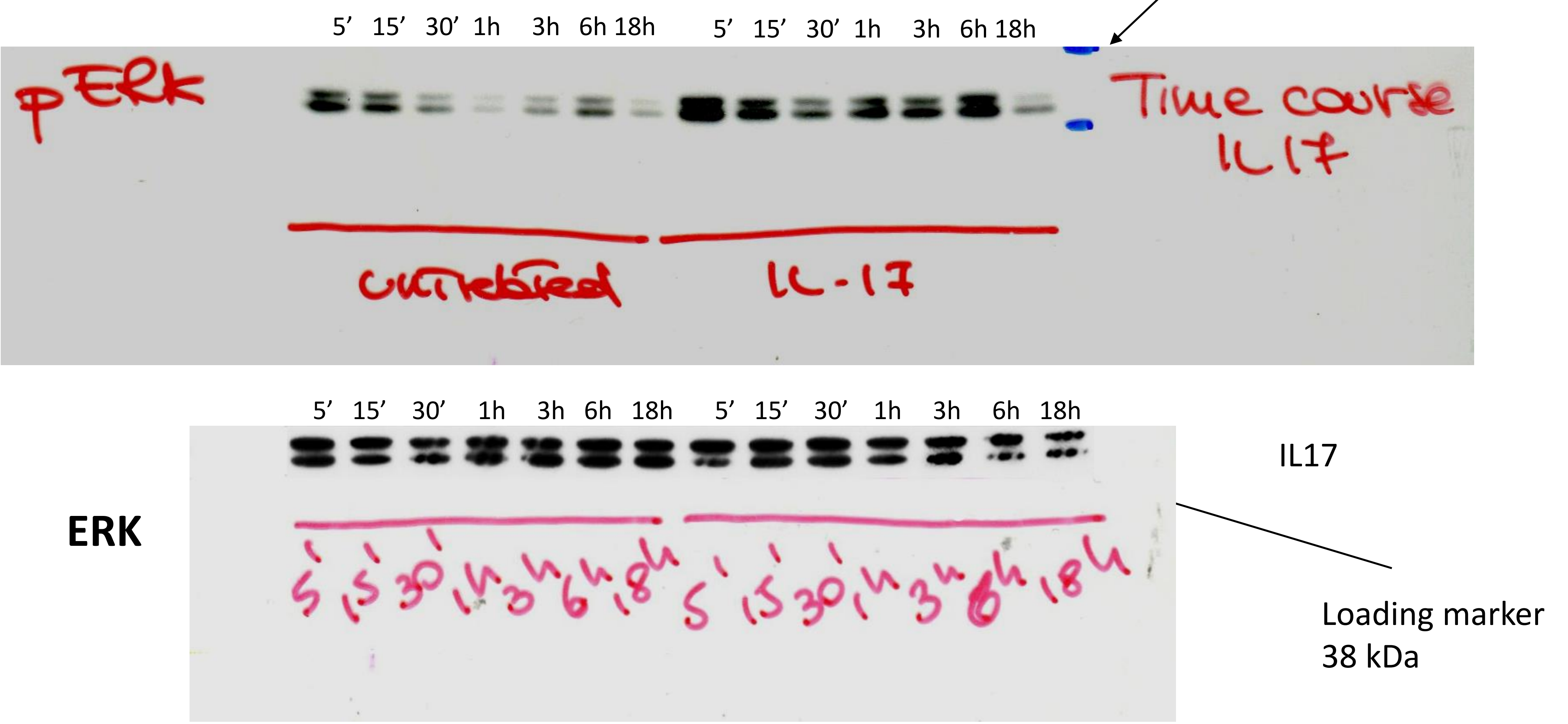

Blots used for <u>Figure 1</u>:

Figure 1 Panel A: HDMEC stimulated or not with 50n/ml of IL17A at different time-point s described in the blots:

p-65 and P65
molecular weight: 86-88 kDa

P-P65
65kDa

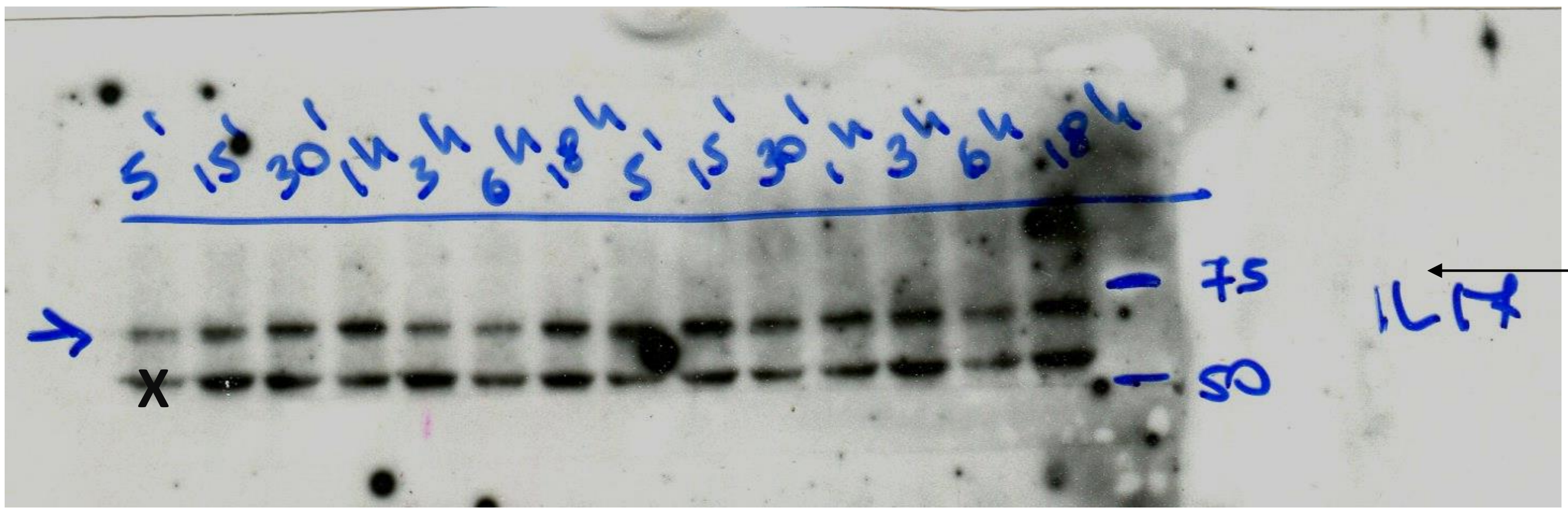

Loading marker
75-50KDa

P65
65kDa

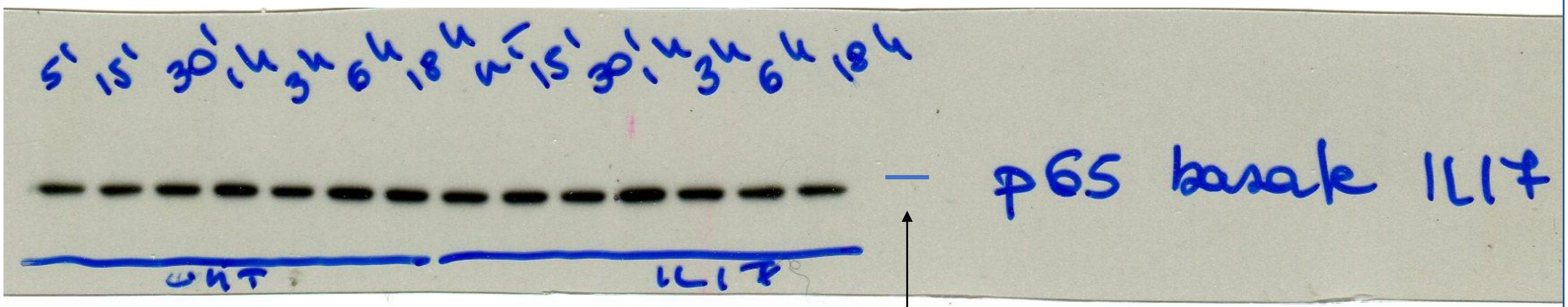

Loading marker
75-50KDa

β-actin
molecular weight 42-45kDa

5’ 15’ 30’ 1h 3h 6h 18h 5’ 15’ 30’ 1h 3h 6h 18h

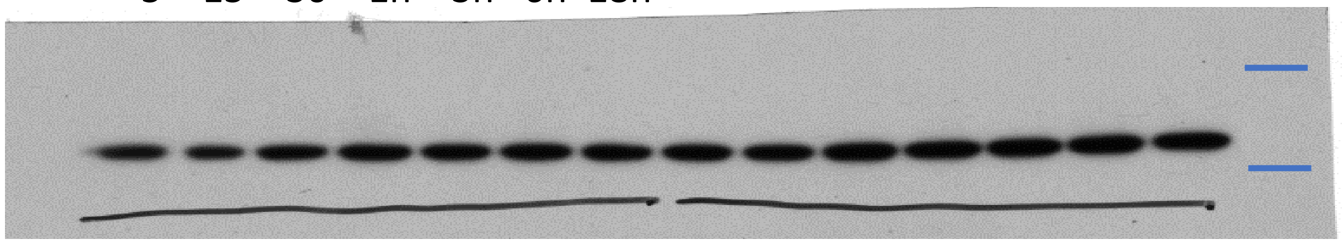

Loading marker
38 kDa and 50 kDa

untreated IL17

Blots used for **<u>Figure</u> 1**:

Figure 1 Panel B: HDMEC stimulated or not with 50n/ml of IL36γ at different time-point as described in the blots:

p-STAT3 and STAT3
molecular weight: 86-88 kDa

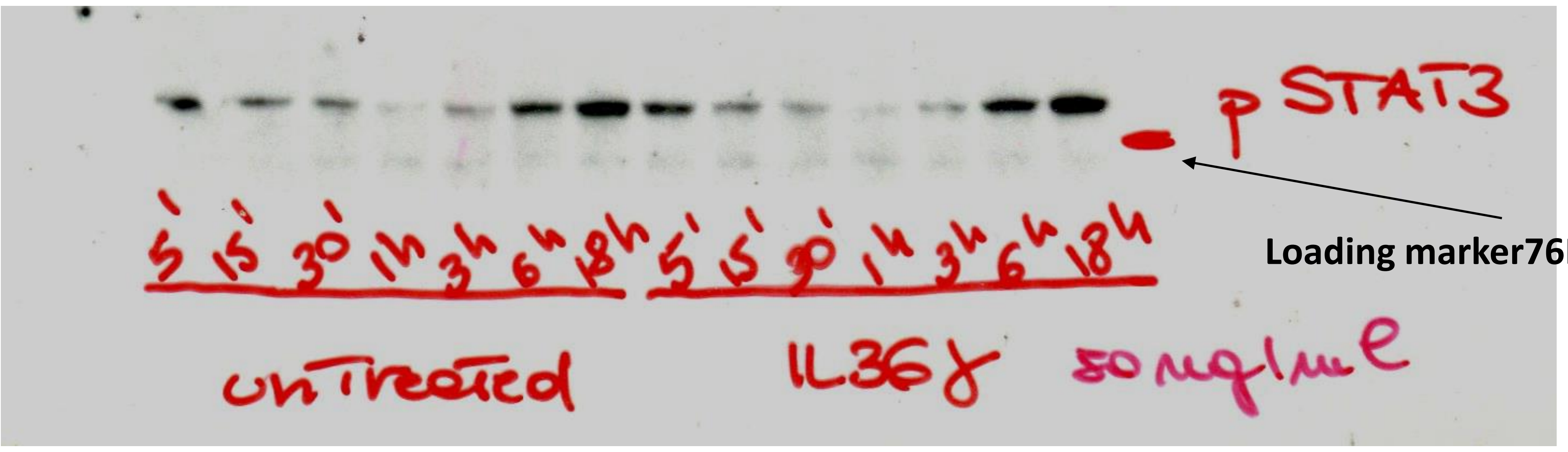

Loading marker76KDa

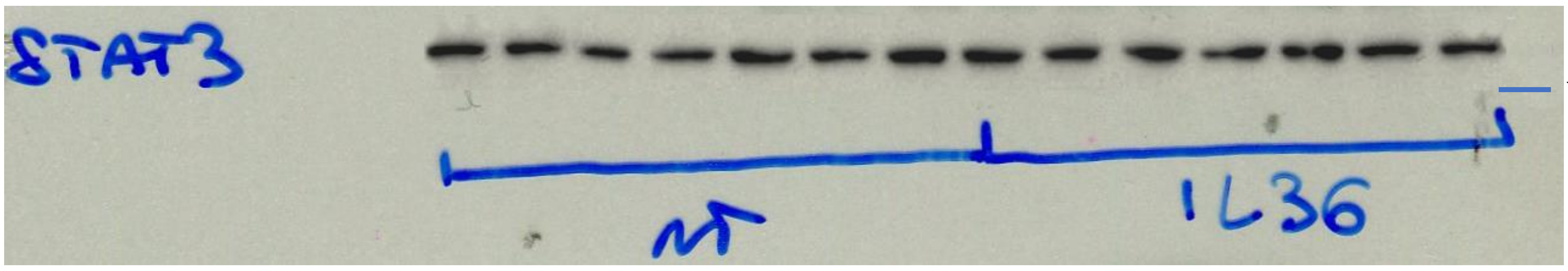

Loading marker 76KDa

p-ERK and ERK
molecular weight 42-44 kDa

Loading marker 50-38 kDa

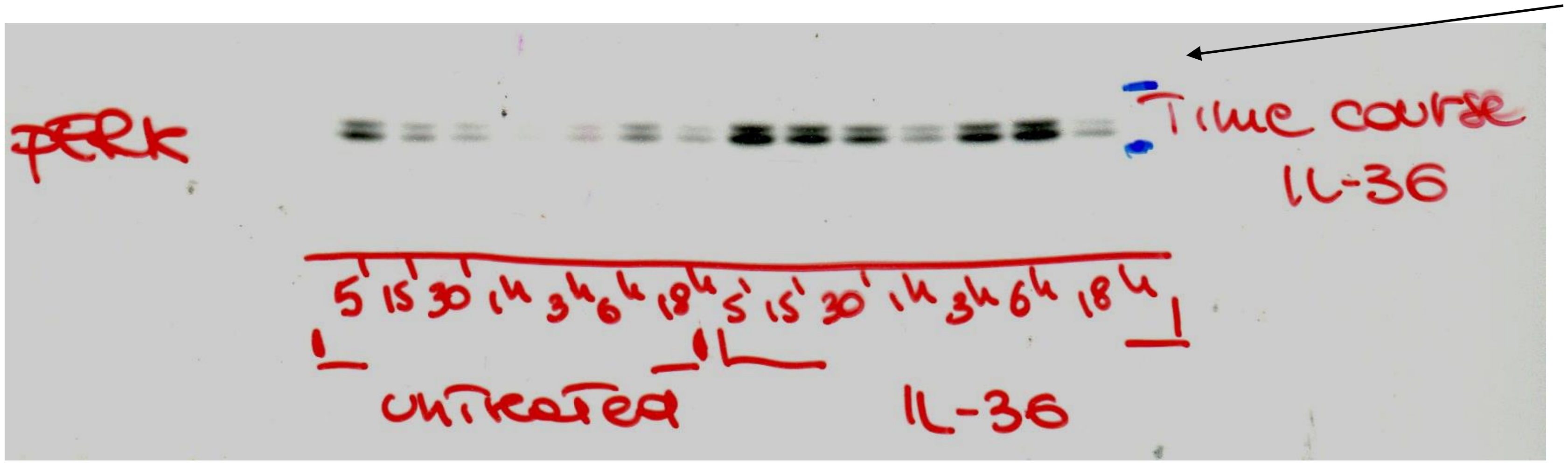

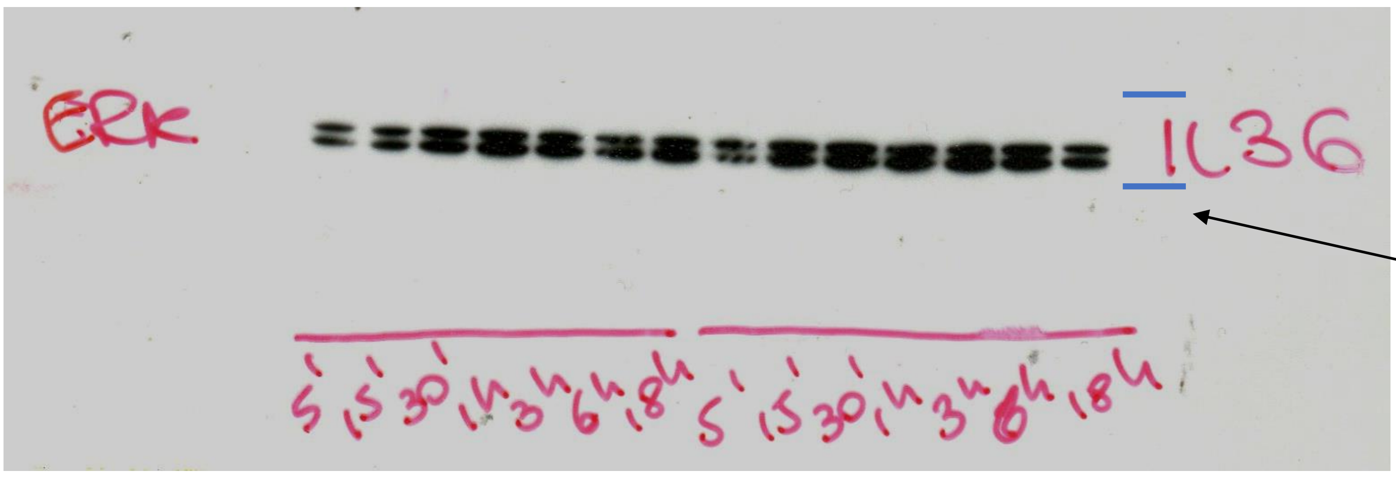

Loading marker 50-38 kDa

Blots used for Figure 1:

Figure 1 Panel B
HDMEC stimulated or not with 50ng/ml of IL36γ at different time-points as described in the following blots:

p-65 and P65
molecular weight: 86-88 kDa

5’ 15’ 30’ 1h 3h 6h 18h 5’ 15’ 30’ 1h 3h 6h 18h

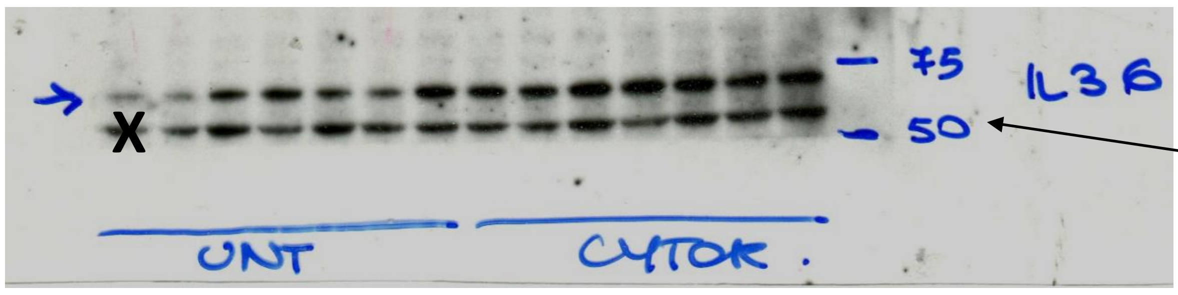

Loading marker
50 and 75 KDa

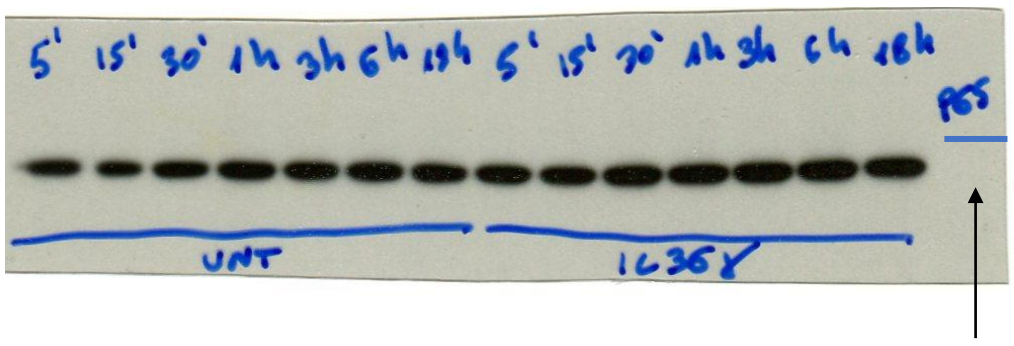

Loading marker
75 KDa

β-actin
molecular weight 42-45kDa

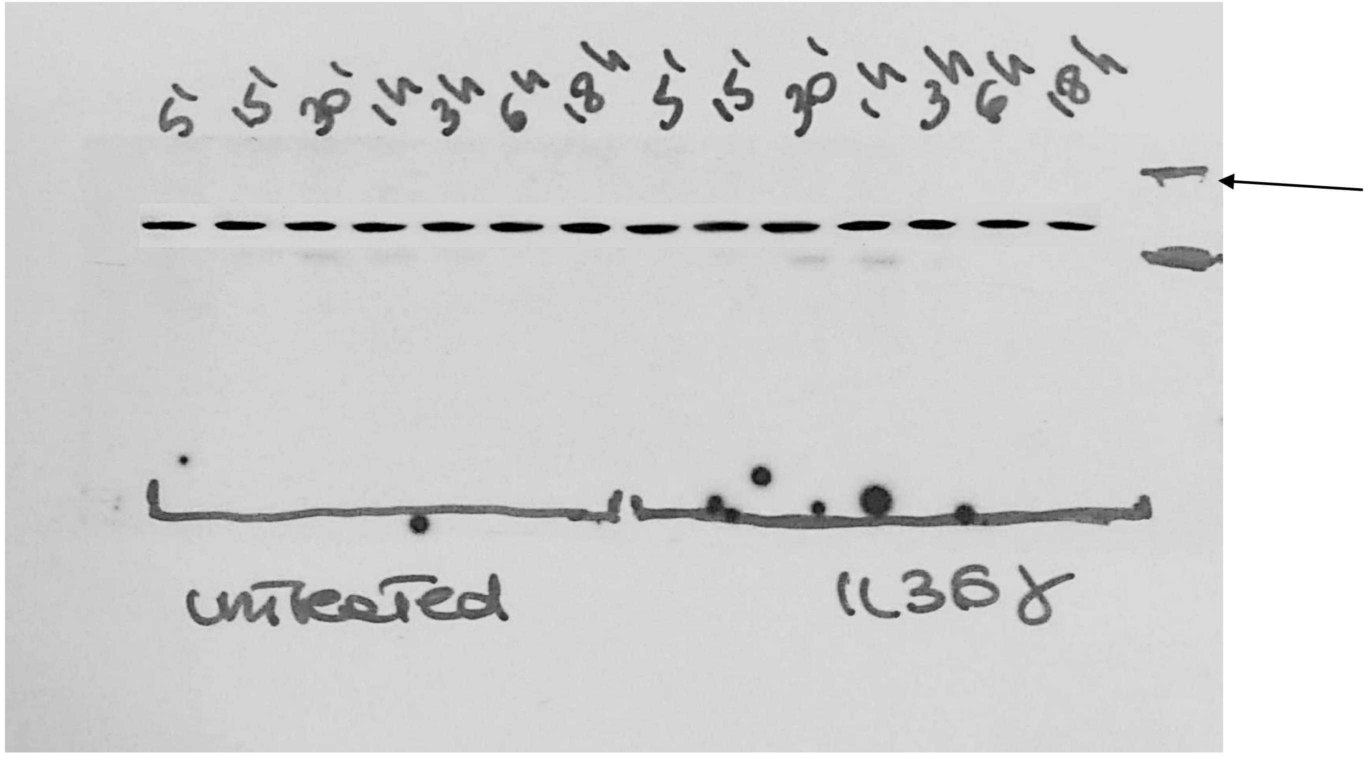

Loading marker
38 kDa and 50 kDa
